# *Plasmodium* Merozoite TRAP Family Protein Is Essential for Vacuole Membrane Disruption and Gamete Egress from Erythrocytes

**DOI:** 10.1016/j.chom.2016.10.015

**Published:** 2016-11-09

**Authors:** Daniel Y. Bargieri, Sabine Thiberge, Chwen L. Tay, Alison F. Carey, Alice Rantz, Florian Hischen, Audrey Lorthiois, Ursula Straschil, Pallavi Singh, Shailja Singh, Tony Triglia, Takafumi Tsuboi, Alan Cowman, Chetan Chitnis, Pietro Alano, Jake Baum, Gabriele Pradel, Catherine Lavazec, Robert Ménard

**Affiliations:** 1 Malaria Biology and Genetics Unit, Pasteur Institute, Paris 75015, France; 2 Department of Parasitology, University of São Paulo-USP, São Paulo 05508-000, SP, Brazil; 3 Department of Life Sciences, Imperial College London, South Kensington, London SW7 2AZ, UK; 4 Department of Pathology, Massachusetts General Hospital, Boston, MA 02114, USA; 5 Division of Cellular and Applied Infection Biology, Institute of Zoology, RWTH Aachen University, Aachen 52074, Germany; 6 Inserm U1016, CNRS UMR 8104, Université Paris Descartes, Institut Cochin, Paris 75014, France; 7 Malaria Parasite Biology and Vaccines Unit, Pasteur Institute, Paris 75015, France; 8 The Walter and Eliza Hall Institute of Medical Research, Parkville 3052, VIC, Australia; 9 Department of Medical Biology, University of Melbourne, Parkville 3052, VIC, Australia; 10 Division of Malaria Research, Proteo-Science Center, Ehime University, Matsuyama, Ehime 790-8577, Japan; 11 Dipartimento di Malattie Infettive, Parassitarie ed Immunomediate, Istituto Superiore di Sanità, Rome 00161, Italy

## Abstract

Surface-associated TRAP (thrombospondin-related anonymous protein) family proteins are conserved across the phylum of apicomplexan parasites. TRAP proteins are thought to play an integral role in parasite motility and cell invasion by linking the extracellular environment with the parasite submembrane actomyosin motor. Blood stage forms of the malaria parasite *Plasmodium* express a TRAP family protein called merozoite-TRAP (MTRAP) that has been implicated in erythrocyte invasion. Using MTRAP-deficient mutants of the rodent-infecting *P. berghei* and human-infecting *P. falciparum* parasites, we show that MTRAP is dispensable for erythrocyte invasion. Instead, MTRAP is essential for gamete egress from erythrocytes, where it is necessary for the disruption of the gamete-containing parasitophorous vacuole membrane, and thus for parasite transmission to mosquitoes. This indicates that motor-binding TRAP family members function not just in parasite motility and cell invasion but also in membrane disruption and cell egress.

## Highlights

• Merozoite TRAP protein, MTRAP, is dispensable for *Plasmodium* asexual blood stages
• MTRAP-deficient parasites are blocked from transmission to mosquitoes
• MTRAP is expressed in *Plasmodium* sexual stages and is essential for gamete egress
• MTRAP-deficient gametes fail to lyse the parasitophorous vacuole membrane for egress

## Graphical Abstract

**Figure:**
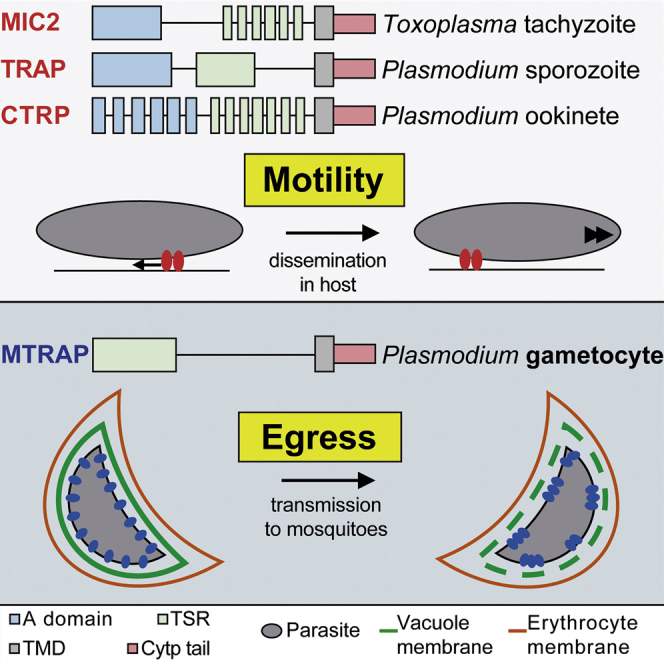

## Teaser

MTRAP, a protein expressed in *Plasmodium* blood stages, was thought to function during invasion of erythrocytes by the asexual merozoite stage. Bargieri et al. report that MTRAP is dispensable for merozoite invasion but is essential for egress of the gamete sexual stage from erythrocytes and for parasite transmission to mosquitoes.

## Introduction

The cyclic fevers typically associated with malaria are caused by repeated cycles of *Plasmodium* multiplication inside host erythrocytes. During a cycle, which lasts 24–72 hr depending on the *Plasmodium* species, the merozoite form of the parasite invades an erythrocyte inside a vacuole where it transforms into 10–30 new merozoites that eventually egress from the host erythrocyte (Tilley et al., 2011). Instead of multiplying, internalized merozoites can also transform into sexual stages, the gametocytes, which do not divide and circulate until they are ingested by an *Anopheles* mosquito (Tibúrcio et al., 2015). In the mosquito midgut lumen, gametocytes become activated and transform into gametes that rapidly egress from erythrocytes (Wirth and Pradel, 2012). After fertilization and parasite development in the mosquito, a process that takes 2–3 weeks, invasive sporozoites form and are transmitted to a new mammalian host where they transform, inside hepatocytes, into first-generation merozoites (Lindner et al., 2012).

To complete its life cycle, the parasite needs to be motile and to actively invade host cells. With the exception of flagellum-based motility used by male gametes, *Plasmodium* locomotes via a substrate-dependent type of motility called gliding (King, 1988). The ookinete stage (motile zygote) glides in the mosquito midgut lumen and crosses its epithelium (Zieler and Dvorak, 2000), while the sporozoite glides in the mosquito salivary system (Frischknecht et al., 2004) as well as in the skin (Vanderberg and Frevert, 2004) and liver of the mammalian host (Amino et al., 2006). The parasite also needs to invade host cells. Host cell invasion is a process by which the parasite actively enters the target cell inside a parasitophorous vacuole (PV) created by the invagination of the host cell membrane (Aikawa et al., 1978). Only the merozoite and sporozoite forms invade host cells—the erythrocytes and hepatocytes, respectively.

Gliding motility and host cell invasion are both active processes powered by an actomyosin motor. The motor is located in the space that separates the parasite plasma membrane (PPM) and a layer of flattened vesicles called inner-membrane complex (IMC) or alveoli (Gould et al., 2011). The motor comprises a single-headed unconventional myosin of the apicomplexan-specific XIV class, called MyoA, bound to the IMC, and dynamic filaments of actin located underneath the plasma membrane (Heintzelman, 2015). A number of structural proteins called gliding-associated proteins appear to tether MyoA to the IMC as well as hold the PPM and the IMC together (Boucher and Bosch, 2015). Finally, transmembrane proteins link the submembrane motor to the extracellular environment. Their stable interaction with the matrix/host cell surface constitutes an anchor on which myosins pull to move the parasite forward (King, 1988).

To date, the parasite transmembrane proteins that have been identified as links between the parasite motor and the extracellular milieu all belong to the thrombospondin-related anonymous protein (TRAP) family of proteins (Morahan et al., 2009). These proteins are type I transmembrane proteins that share a functionally conserved cytoplasmic tail (Kappe et al., 1999) that binds actin (Jewett and Sibley, 2003), and an ectodomain exposing various ligand-binding modules including a thrombospondin type I repeat (TSR) (Matuschewski et al., 2002). They are specific to the apicomplexan phylum of protists, being expressed, among human pathogens, in *Plasmodium*, *Toxoplasma*, *Babesia*, and *Cryptosporidium*. In *Plasmodium*, the sporozoite stage expresses three members of the family—TRAP; TRAP-related protein (TREP), also called S6; and TRAP-like protein (TLP)—which all play a role in sporozoite gliding on substrates and within tissues (Combe et al., 2009, Heiss et al., 2008, Steinbuechel and Matuschewski, 2009, Sultan et al., 1997). The ookinete stage expresses a single member, called circumsporozoite protein and thrombospondin-related anonymous protein-related protein (CTRP), which is essential for ookinete gliding motility (Dessens et al., 1999).

Merozoite TRAP (MTRAP) is a TRAP family member that was reported as expressed in the merozoite (Baum et al., 2006), which invades erythrocytes but does not exhibit gliding motility. The *mtrap* gene is conserved and syntenic among *Plasmodium* species. In *P. falciparum*, *mtrap* could not be disrupted (Baum et al., 2006), in agreement with the view that MTRAP might be involved in merozoite invasion of erythrocytes. Biochemical approaches found that the *P. falciparum* MTRAP ectodomain bound to the GPI-linked protein semaphorin-7A (CD108) on human erythrocytes (Bartholdson et al., 2012). In this interaction, two MTRAP monomers were proposed to interact via their tandem TSRs with the Sema domains of a Semaphorin-7A homodimer. More recently, the MTRAP cytoplasmic tail was shown to be sufficient to polymerize actin (Diaz et al., 2014). These data all favor a role for MTRAP during merozoite invasion of erythrocytes, possibly acting as a bridge between the motor and the erythrocyte surface.

Here we address the role of MTRAP using rodent-infecting *P. berghei* and human-infecting *P. falciparum* parasites. Results indicate that MTRAP is not critical for merozoite invasion of erythrocytes but is crucial for gamete egress from the PV membrane (PVM) and thus parasite transmission to mosquitoes.

## Results

### MTRAP Is Dispensable for *P. berghei* Asexual Blood Stages

**Figure 1:**
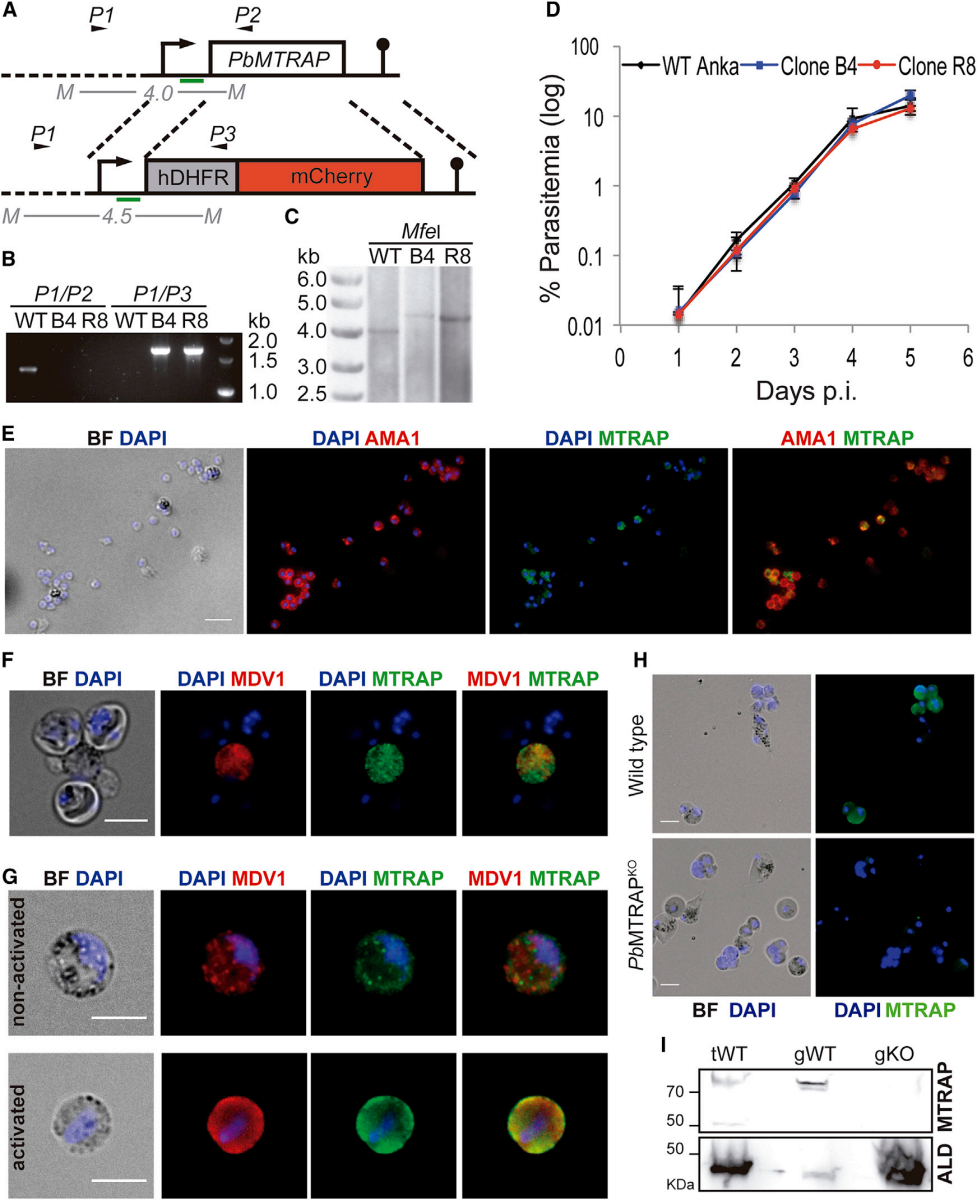
Generation of *Pb*MTRAP^KO^ Clones (A) Illustration of the strategy used for replacing the coding sequence of MTRAP by a cassette for expression of the selection marker human dihydrofolate reductase (hDHFR), that confers resistance to pyrimethamine, and a cassette for expression of mCherry (red fluorescence). The primers (arrowheads) and probes (green bars) used for genotyping are shown. The expected fragment sizes after digestion of the loci with MfeI are also shown. (B) PCR analysis of the *mtrap* locus in wild-type (WT) or mutant (B4 and B8) parasites. P1/P2 pair of primers is specific to the WT locus, and P1/P3 pair is specific to integration of the targeting sequence. (C) Southern blot detecting the *mtrap* locus in wild-type (WT) or mutant (B4 and B8) parasites after digestion of genomic DNA with MfeI. The probe used is illustrated in (A) (green bars). (D) Growth curves assessed daily in mouse blood after infection with wild-type (black line) or the two clones of *Pb*MTRAP^KO^ parasites (blue and red lines). Results are shown as mean ± SD and are representative of three independent experiments. N = 5 for each group. (E) Fluorescence microscopy with anti-*Pb*MTRAP (green), anti-AMA1 (red), and DAPI (blue) in wild-type *P. berghei* merozoites. BF, brightfield. Scale bar, 5 μm. (F) Fluorescence microscopy with anti-*Pb*MTRAP (green), anti-MDV-1/PEG3 (red) and DAPI (blue) in a wild-type *P. berghei* sexual stage isolated from infected mouse blood. BF, brightfield. Scale bar, 5 μm. See also Figure S1. (G) Fluorescence microscopy with anti-*Pb*MTRAP (green), anti-MDV-1/PEG3 (red), and DAPI (blue) in nonactivated or 10 min activated wild-type *P. berghei* sexual stages isolated from infected mouse blood. BF, brightfield. Scale bar, 5 μm. (H) Fluorescence microscopy with anti-*Pb*MTRAP (green) and DAPI (blue) in MTRAP knockout (*Pb*MTRAP^KO^) and wild-type parasites. BF, brightfield. Scale bar, 5 μm. (I) Western blot analysis of the gametocyte extract of *Pb*MTRAP^KO^ (gKO) with a specific antibody recognizing the MTRAP C-terminal region. Total extract (tWT) or gametocyte extract (gWT) of wild-type *P. berghei* ANKA strain was used as control. Anti-aldolase (ALD) was used as loading control. The anti-MTRAP recognizes two specific bands in tWT and one specific band in gWT parasites. No bands are recognized in the three gKO extract.

We first investigated the role of MTRAP using the rodent-infecting *P. berghei* model. *mtrap* knockout (*Pb*MTRAP^KO^) clones, B4 and R8, were derived from WT *P. berghei* ANKA by replacing the full *mtrap* coding sequence by two cassettes expressing resistance to pyrimethamine or the red fluorescent protein mCherry (Figures 1A–1C). Intravenous injection of *Pb*MTRAP^KO^ or WT parasites in mice resulted in identical parasite growth curves, i.e., an ∼10-fold daily increase in parasitemia during exponential multiplication (Figure 1D). The absence of any detectable effect of *mtrap* deletion on blood stage parasite growth thus raised the hypothesis that MTRAP does not function at that stage.

Isolated blood stages were then analyzed by immunofluorescence assays (IF) using a polyclonal antibody generated against a peptide sequence from the cytoplasmic tail of *P. berghei* MTRAP. In WT parasites, only a proportion (48% ± 12.2%) of merozoites, defined by positive staining of apical membrane antigen 1 (AMA1), displayed a positive MTRAP signal (Figure 1E), differently from previous findings in *P. falciparum*, in which all merozoites are MTRAP positive (Riglar et al., 2016). MTRAP staining was predominantly associated with sexual stages of the parasite (Figures 1F–1H). Isolated *P. berghei* gametocytes, identified by staining male development-1 (MDV-1)/protein of early gametocyte 3 (PEG3) in osmiophilic bodies (Hayton and Templeton, 2008), exhibited a punctate MTRAP staining (Figure 1F) with asexual trophozoite stages serving as negative controls. The punctate MTRAP staining in nonactivated *P. berghei* gametocytes did not colocalize with MDV-1/PEG3, and after activation of the gametocytes for 10 min it became more diffuse and mostly peripheral (Figures 1G and S1). MTRAP was not detected in any *Pb*MTRAP^KO^ parasite population by immunofluorescence (IF) (Figure 1G) or by western blot (Figure 1H).

### PbMTRAP^KO^ Are Blocked in Mosquito Transmission

**Figure 2:**
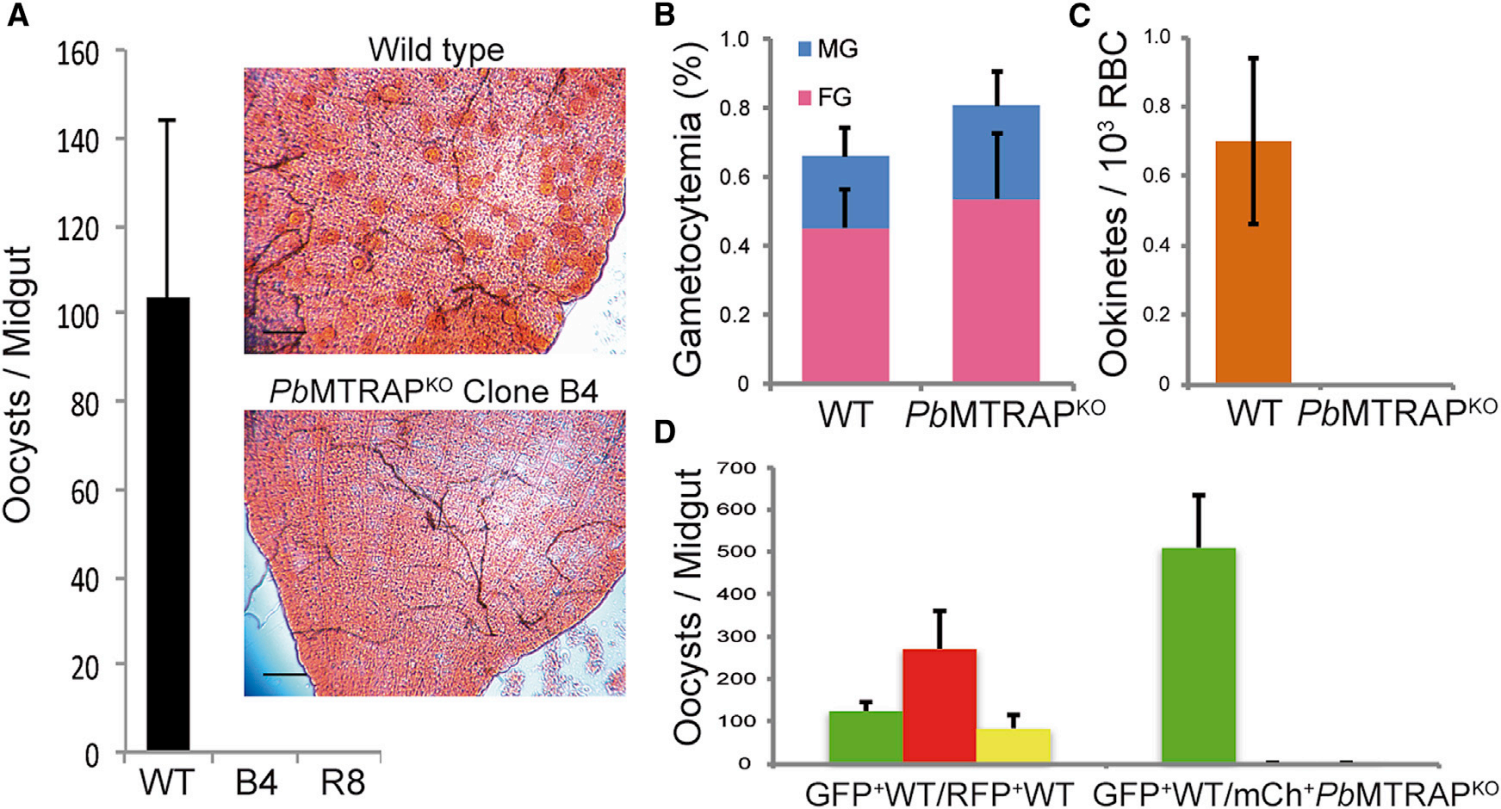
*Pb*MTRAP^KO^ Are Blocked in Mosquito Transmission (A) *P. berghei* oocysts in the midgut of mosquitoes fed onto mice infected with wild-type or *Pb*MTRAP^KO^. Oocysts are visualized by mercurochrome staining of mosquito midguts 7 days after mosquito feeding. Scale bar, 100 μm. Quantification is shown on the left. N = 100 mosquitoes for each group. (B) Quantification of *P. berghei* male gametocytes (MG, blue) and female gametocytes (FG, pink) circulating in mouse blood infected with either wild-type (WT) or *Pb*MTRAP^KO^ parasites. (C) Quantification of in vitro ookinete formation from gametocytes circulating in mouse blood infected with either wild-type (WT) or *Pb*MTRAP^KO^ parasites. (D) Quantification of green, red, and yellow (green + red) *P. berghei* oocyst numbers by fluorescence microscopy of mosquito midguts 7 days after mosquito feeding onto mice infected with a control mixture of green and red wild-type parasites (GFP^+^WT and RFP^+^WT, respectively), or with a mixture of *Pb*MTRAP^KO^ (red, mCh^+^*Pb*MTRAP^KO^) and wild-type green (GFP^+^WT) parasites. N = 100 mosquitoes for each group. The gametocytemia of green and red parasites were comparable in infected mice of the different groups used for mosquito feeding (data not shown). For all panels, data are shown as mean ± SD and are representative of three independent experiments.

To test whether MTRAP might play a role in sexual stages, *Anopheles stephensi* mosquitoes were blood fed on mice infected with either GFP^+^WT *P. berghei* ANKA or the mCherry^+^*Pb*MTRAP^KO^ clones, and the numbers of oocysts formed in mosquito midguts were counted 7 days postfeeding. While mosquitoes feeding on WT-infected mice consistently infected more than 70% of mosquitoes with over 100 oocysts per midgut on average, *Pb*MTRAP^KO^ oocysts were not observed (Figure 2A). The inability to infect mosquitoes was not due to impaired gametocytogenesis, since mice infected with *Pb*MTRAP^KO^ had normal numbers of circulating male and female gametocytes (Figure 2B) that were morphologically normal as judged by Giemsa staining (data not shown). However, when *Pb*MTRAP^KO^ gametocytes were activated in vitro and allowed to fertilize, ookinetes, the motile zygote stage, were not formed (Figure 2C). These data indicated a major role for MTRAP in a step following gametocyte activation.

To date, several *Plasmodium* products have been shown to play a role during the sexual phase of the parasite life cycle, which include the mitogen-activated kinase 2 (map-2) (Rangarajan et al., 2005, Tewari et al., 2005), actin-II (Deligianni et al., 2011), the *Plasmodium* perforin-like protein 2 (PPLP2) (Deligianni et al., 2013, Wirth et al., 2014), Pfg377 (de Koning-Ward et al., 2008), MDV-1/ PEG3 (Ponzi et al., 2009), and the gamete egress and sporozoite traversal (GEST) protein (Talman et al., 2011). Using gene targeting in *P. falciparum*, GEST, MDV-1/PEG3, and PPLP2 were found to be important for both male and female gametocytes, while using gene targeting in *P. berghei*, map-2, PPLP2, and actin-II were reported to cause male-specific phenotypes.

To test whether MTRAP function is gender specific, mosquitoes were fed on mice coinfected with GFP^+^WT (green) and mCherry^+^*Pb*MTRAP^KO^ (red) parasites, and oocysts formed in mosquito midguts were counted 7 days after feeding. As expected, mosquitoes fed on mice infected with a control mixture of GFP^+^WT and RFP^+^WT parasites had midguts infected with green, red, or yellow oocysts (Figure 2D). In contrast, mosquitoes fed on mice infected with GFP^+^WT and mCherry^+^*Pb*MTRAP^KO^ parasites had midguts infected with green oocysts only (Figure 2D). This demonstrated that neither male nor female *Pb*MTRAP^KO^ gametocytes could fertilize their WT green counterparts and therefore that MTRAP is important for both male and female gametocyte development.

### PbMTRAP^KO^ Gametes Are Trapped inside the PV Membrane

**Figure 3:**
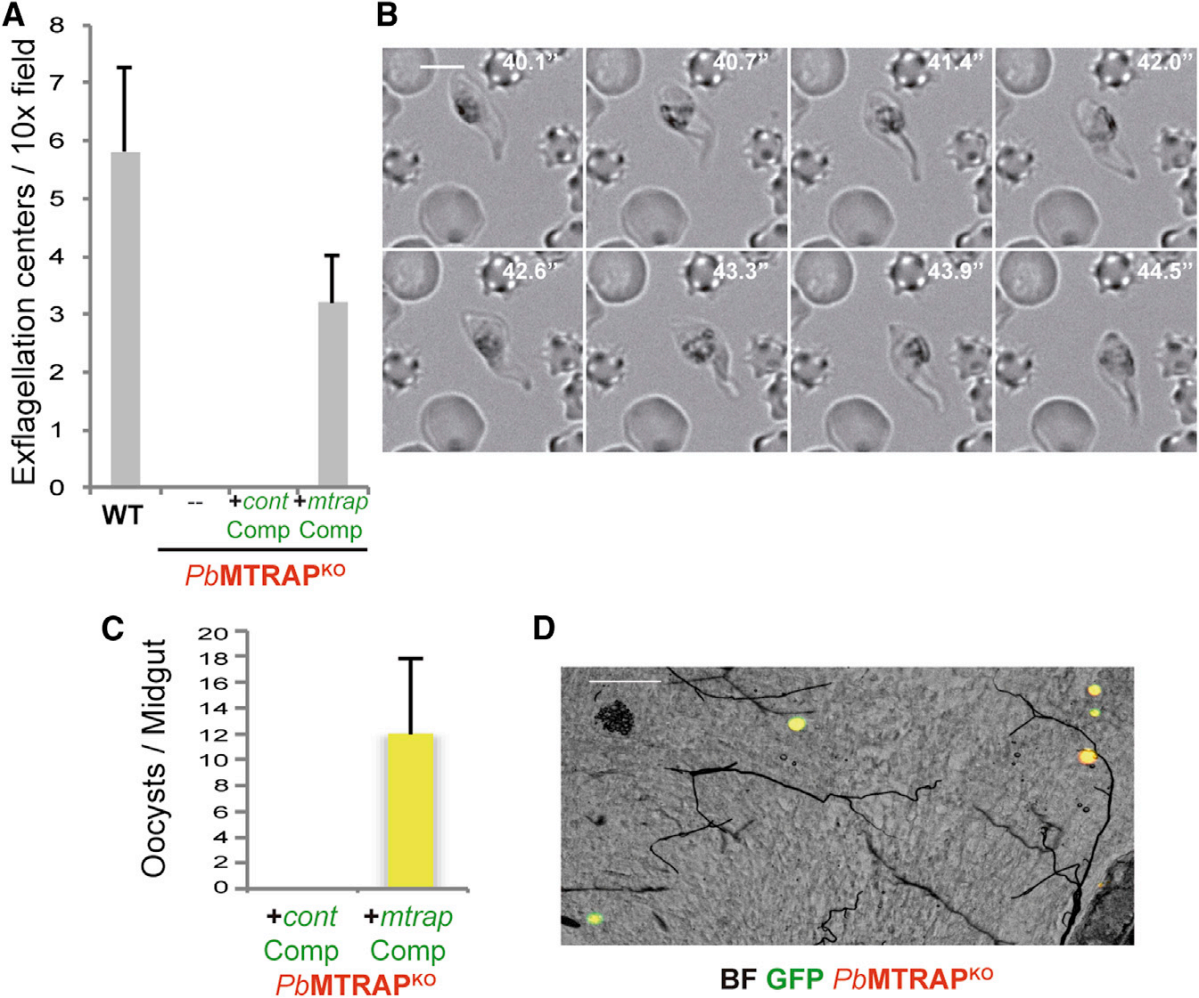
*Pb*MTRAP^KO^ Male Gametocytes Do Not Make Exflagellation Centers but Form Motile Axonemes (A) Quantification of exflagellation centers per 10× field formed by in vitro-activated wild-type *P. berghei* (WT), *Pb*MTRAP^KO^ male gametocytes, or *Pb*MTRAP^KO^ carrying either a control episome (*cont*Comp) or an episome with the promoter and coding sequence of *mtrap* cloned upstream the 3′UTR of *trap*, a centromeric sequence and a cassette for GFP (green) expression (*mtrap*Comp). The results are shown as mean ± SD and are representative of four independent experiments. (B) Time-lapse microscopy of an activated *Pb*MTRAP^KO^ male gametocyte. The time in seconds is shown in each image. The results are representative of five independent experiments. (C) Quantification of oocysts per midgut of mosquitoes fed onto mice infected with *Pb*MTRAP^KO^ carrying either the control episome (*cont*Comp) or the episome with *mtrap* (*mtrap*Comp). The results are shown as mean ± SD and are representative of two independent experiments. (D) Fluorescence microscopy of mosquito midgut 7 days after mosquito feeding onto mice infected with *Pb*MTRAP^KO^ (red) electroporated with the *mtrap*Comp episome. The presence of the episome is depicted by green fluorescence, and parasites are red fluorescent. Single color oocysts were never seen. N = 100 mosquitoes. Scale bar, 100 μm.

*P. berghei* gamete formation and host cell egress is readily observable in vitro. While a female gametocyte forms a single macrogamete, the male gamete undergoes three mitotic divisions, assembles eight intracytoplasmic axonemes, and produces eight flagellated microgametes in just 10–15 min (Guttery et al., 2015). “Exflagellation” occurs when male microgametes use microtubule-based movements to leave the erythrocyte host and bind egressed macrogametes, and exflagellation centers (ECs), made of gametes attached to erythrocytes, can be readily observed by microscopy. While around five ECs per 10× microscopy field were counted upon WT gametocyte activation in vitro, activated *Pb*MTRAP^KO^ gametocytes did not form ECs (Figure 3A), even when gametocytemia in the mouse blood was as high as 1%. Activated male *Pb*MTRAP^KO^ gametocytes formed motile flagella, but they remained intracellular, beating as a thick bundle of flagella, suggesting that they were unable to egress from the PV or the host erythrocyte (Figure 3B; Movie S1). A similar conclusion of lack of egress of *P. berghei* MTRAP^KO^ was reported in a recently published paper (Kehrer et al., 2016).

**Figure 4:**
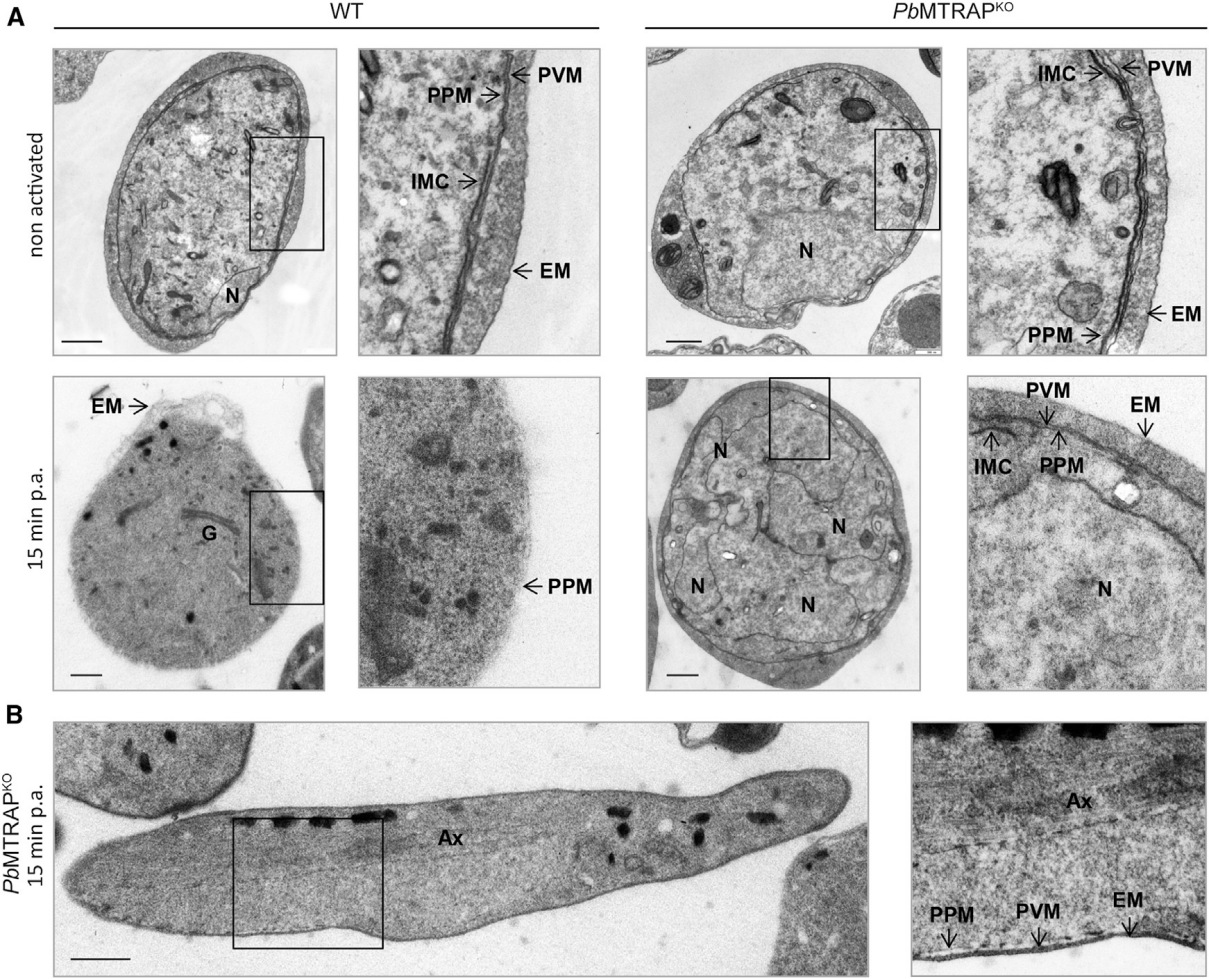
*Pb*MTRAP^KO^ Gametes Are Trapped inside the PV Membrane (A) Micrographs of wild-type *P. berghei* (WT) or *Pb*MTRAP^KO^ gametocytes isolated from infected mice blood and immediately fixed (nonactivated) or fixed after activation in vitro for 15 min (15 min p.a.) in ookinete medium. Ultrastructures are indicated with arrows. IMC, inner membrane complex; PPM, parasite plasma membrane; PVM, parasitophorous vacuole membrane; EM, erythrocyte membrane; N, nucleus; G, Golgi complex. Results are representative of three independent experiments. N = 6 for WT and 19 for *Pb*MTRAP^KO^. Scale bars, 1 μm. (B) Micrograph of a male *Pb*MTRAP^KO^ gametocyte activated in vitro for 15 min in ookinete medium. Ultrastructures are shown as in (A), except for Ax, axonemes. Scale bars, 1 μm.

Gametes are formed in the first minutes of activation. Within 5 min, the PVM is disrupted, leaving the gametes surrounded by the erythrocyte membrane (EM), which is lysed after another 10 min (Sologub et al., 2011). To further study the *Pb*MTRAP^KO^ gametes, purified *Pb*MTRAP^KO^ or WT gametocytes from the blood of infected mice were fixed without activation or postactivation following 15 min incubation in ookinete medium and analyzed by electron microscopy. Both nonactivated WT and *Pb*MTRAP^KO^ gametocytes were found surrounded by three enveloping membranes, i.e., the EM, the PVM, and the PPM. Underneath the PPM, the double membrane of the IMC was in most cases visible (Figure 4A). Following activation, WT gametes were devoid of surrounding host membranes, while the *Pb*MTRAP^KO^ male and female gametes were consistently wrapped in intact PVM and EM (Figure 4A). Male *Pb*MTRAP^KO^ gametes with formed axonemes could be observed surrounded by PV and host cell membranes (Figure 4B), explaining the observations made by live microscopy (Figure 3B). We conclude that *Pb*MTRAP^KO^ gametocytes can be activated and initiate gametogenesis, but the lack of MTRAP blocks *P. berghei* gamete egress because of an inability to disrupt the membrane of the PV.

### Complementation of PbMTRAP^KO^

To verify that the *Pb*MTRAP^KO^ phenotype was specifically due to lack of MTRAP, we attempted to complement the defective mutants. The promoter and coding sequence of *P. berghei mtrap* were fused to the 3′UTR of *trap* in a plasmid containing a cassette constitutively expressing GFP and a centromeric sequence, PbCEN5-core, conferring plasmid stability by even segregation during schizogony (Iwanaga et al., 2010). *Pb*MTRAP^KO^ blood stages electroporated with the plasmid were injected into mice and after 2 days, GFP-expressing, episome-bearing parasites were FACS sorted. An expanded, sorted parasite population containing ∼65% of GFP^+^ individuals was passaged to mosquitoes and oocysts were examined in mosquito midguts 7 days after feeding. Whereas *Pb*MTRAP^KO^ parasites carrying a control episome lacking *mtrap* did not form ECs after in vitro activation and remained blocked in transmission like *Pb*MTRAP^KO^ parasites, FACS-sorted parasites were able to form ECs (Figure 3A) and oocysts in the midgut of mosquitoes, which were all yellow (Figures 3C and 3D). Therefore, *mtrap* expression in the *Pb*MTRAP^KO^ partially (55% in vitro and 12% in vivo) but specifically restored normal parasite transmission.

### MTRAP Is Dispensable for *P. falciparum* Asexual Blood Stages

**Figure 5:**
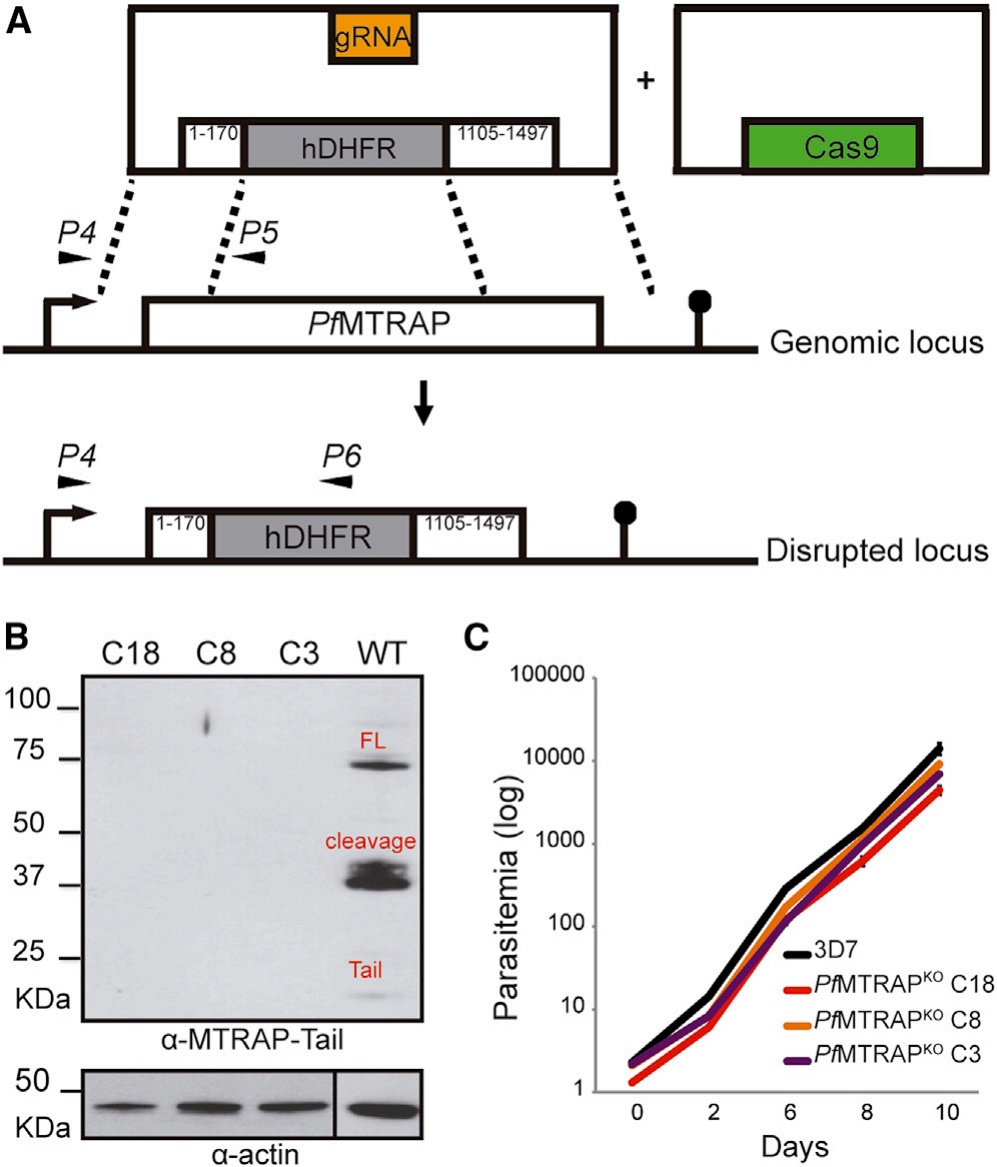
MTRAP Is Dispensable for *P. falciparum* Asexual Stages (A) Illustration of the strategy used to target *P. falciparum mtrap* for disruption. Two plasmids were transfected in the *P. falciparum* 3D7 strain, one plasmid carrying a guide DNA sequence (GAATGGTCAGAATGTAAAGA) and a hDHFR cassette flanked by two homology regions with the 5′and 3′ sequences of the *mtrap* coding sequence as indicated in the figure, and the second plasmid bearing a cassette for Cas9 expression. Double homologous recombination replaces 935 base pairs of the *mtrap* coding sequence by the hDHFR cassette, creating a disrupted locus. Primers used for PCR specific detection of the genomic or the disrupted loci are shown. See also Figure S2. (B) Western blot analysis of the *Pf*MTRAP^KO^ clones C3, C8, and C18 with a specific antibody recognizing the MTRAP C-terminal region (α-MTRAP-Tail). Wild-type *P. falciparum* 3D7 strain (WT) was used as control. The α-MTRAP-Tail recognizes three specific bands in WT parasites, FL as the full-length protein, cleavage as a processed fragment, and Tail as the C-terminal region after processing. No bands are recognized in the three *Pf*MTRAP^KO^ clones. Actin (α-actin) was used as loading controls. (C) Growth curves assessed every 48 hr by flow cytometry in blood cultures of *P. falciparum* wild-type (3D7, black line) or the three *Pf*MTRAP^KO^ clones (colored lines). The experiment was performed in triplicate and the data are presented as mean ± SD.

In view of these data in *P. berghei*, we reattempted to disrupt *mtrap* in *P. falciparum*, this time using the CRISPR-Cas9 technology (Ghorbal et al., 2014). The first 170 bp and the last 392 bp of the *mtrap* coding sequence, along with up- or downstream noncoding regions, were used as homology arms flanking a WR99210-resistance cassette, and the resulting plasmid was transfected into 3D7 blood stages with a Cas9-expressing plasmid (Figure 5A). Double crossover recombination was induced by chromosomal locus disruption by Cas9 and guided by gRNA sequences.

Resistant *P. falciparum* blood stages were detected growing in culture within 14–21 days posttransfection. Three independent clones, C3, C8, and C18, were shown by PCR with mutant- or WT-specific primers (Figure 5A) to have a disrupted *mtrap* locus (Figure S2). To confirm successful *mtrap* disruption, blood stage protein extracts from the three *Pf*MTRAP^KO^ clones were analyzed by western blot using a specific anti-*Pf*MTRAP antibody against the C-terminal region of the protein (α-MTRAP-Tail) (Riglar et al., 2016). The antibody specifically recognized three bands in WT *P. falciparum* 3D7 blood stage extracts corresponding to the full-length (FL) and processed (cleavage and tail) MTRAP, while no specific band was recognized in the protein extracts of the three *Pf*MTRAP^KO^ clones (Figure 5B). Furthermore, a comparison of the in vitro growth of the three *Pf*MTRAP^KO^ clones with WT parasites showed identical asexual growth (Figure 5C). Therefore, as in *P. berghei*, deletion of *mtrap* in *P. falciparum* has no noticeable impact on merozoite invasion, multiplication, or egress. The selection of *Pf*MTRAP^KO^ mutants was reproduced independently in two different laboratories with different transfection plasmids (data not shown) using the CRISPR-Cas9 system.

### MTRAP Is Expressed in the Gametocyte Stages of *P. falciparum*

**Figure 6:**
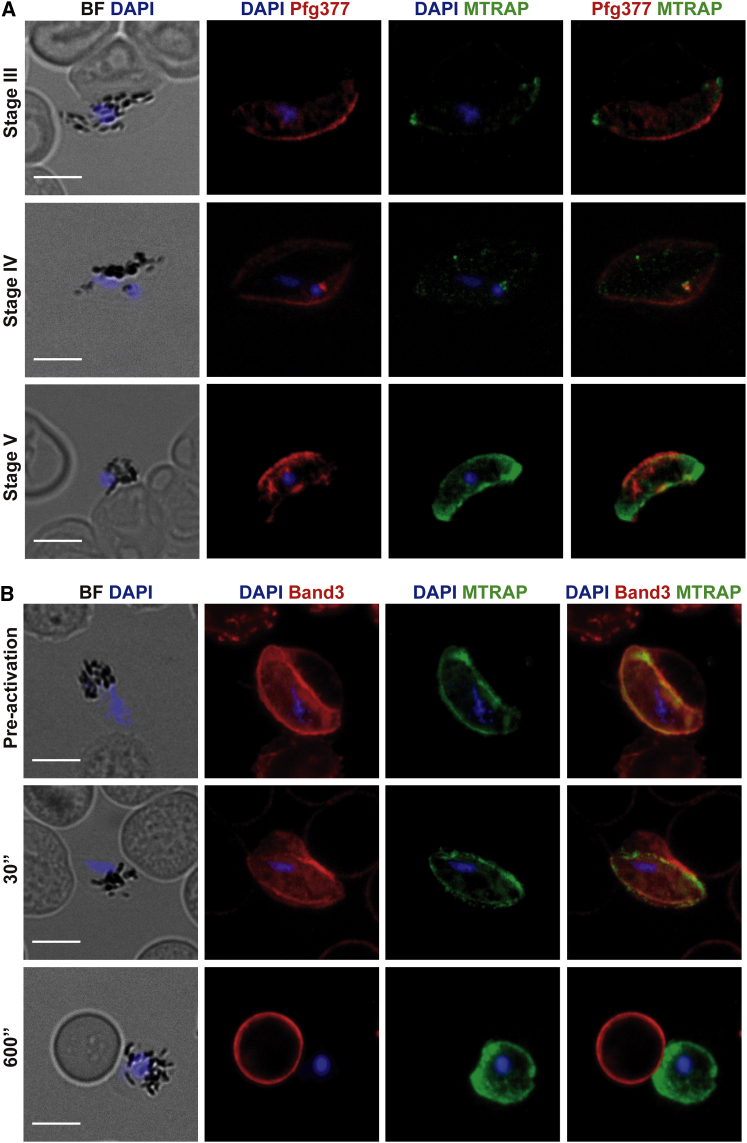
MTRAP Is Expressed in Sexual Stages of *P. falciparum* (A) Fluorescence microscopy with anti-*Pf*MTRAP (green), anti-Pfg377 (red), and DAPI (blue) in wild-type *P. falciparum* sexual stages matured in vitro. Stages III, IV, and V gametocytes are shown. BF, brightfield. Scale bar, 5 μm. See also Figure S3. (B) Fluorescence microscopy with anti-*Pf*MTRAP (green), anti-Band3 (red), and DAPI (blue) in wild-type *P. falciparum* sexual stages matured in vitro. A gametocyte nonactivated (preactivation), a gametocyte activated for 30 s in vitro, and an egressed gamete after 600 s of activation in vitro are shown. BF, brightfield. Scale bar, 5 μm. See also Figure S7A.

To verify MTRAP expression in *P. falciparum* blood stages, new anti-MTRAP antibodies were generated in rabbits immunized with a full-length recombinant *P. falciparum* MTRAP construct expressed by wheat germ cell-free in vitro translation system (Tsuboi et al., 2008). As in *P. berghei*, MTRAP was strongly detected in the sexual stages. When synchronous *P. falciparum* gametocytes at stages III, IV, or V of maturation were analyzed by immunofluorescence assay, MTRAP was detected predominantly in stage V gametocytes and only weakly in stages III and IV (Figures 6A and S3), showing that MTRAP expression increases during gametocyte maturation in *P. falciparum*.

MTRAP was then immunostained in stage V gametocytes along with other relevant sexual stage proteins. MTRAP staining did not colocalize with Pfg377 (Figure 6A) or with DPAP2, a gametocyte-specific *P. falciparum* homolog of dipeptidyl aminopeptidases (Figure S4), which have been both localized to the osmiophilic bodies (OBs) (Suárez-Cortés et al., 2016). This suggested that either MTRAP is not an OB protein or that it is stored a unique OB subset. Costaining with Pfs230, a sexual stage surface antigen, confirmed the peripheral staining of MTRAP (Figures S5A and S5B). Importantly, costainings using antibodies recognizing the MTRAP TSR or tail (Baum et al., 2006, Riglar et al., 2016) and anti-GAP45 (Figures S6A and S6B), a component of the parasite gliding motor, supported a localization in mature gametocytes consistent with the IMC.

*Plasmodium* gametocyte activation and gamete formation comprise a stepwise event during which the PVM is lysed before the host EM (Kuehn and Pradel, 2010). The pattern of MTRAP staining was thus followed over time during *P. falciparum* gametocyte activation. Prior to activation, crescent-shaped gametocytes, displaying peripheral MTRAP staining, were surrounded by an intact EM labeled with anti-Band3 antibody (Figure 6B, top line). After 30 s of activation, gametocytes started rounding up, while remaining surrounded by an intact EM (Figure 6B, middle line). Notably, after 30 s, MTRAP staining appeared patchier than the homogeneous staining prior to activation (Figure 6B, middle line; Figure S7A), suggesting that MTRAP intramembrane displacement might be an early event during gametocyte activation. After 600 s of activation, egressed female gametes appeared spherical and Band3 staining was no longer visible, indicating an extracellular position (Figure 6B, bottom line). Importantly, MTRAP was still detected on these egressed gametes, demonstrating that MTRAP is associated with parasite membrane rather than PVM. Independent labeling using the anti-MTRAP TSR or the anti-MTRAP Tail antibodies over time during gametocyte activation confirmed that the protein is membrane associated in both male and female gametes, displaying punctate labeling foci at the tips of egressed males (Figures S7B and S7C).

### PfMTRAP^KO^ Gametes Fail to Egress

**Figure 7:**
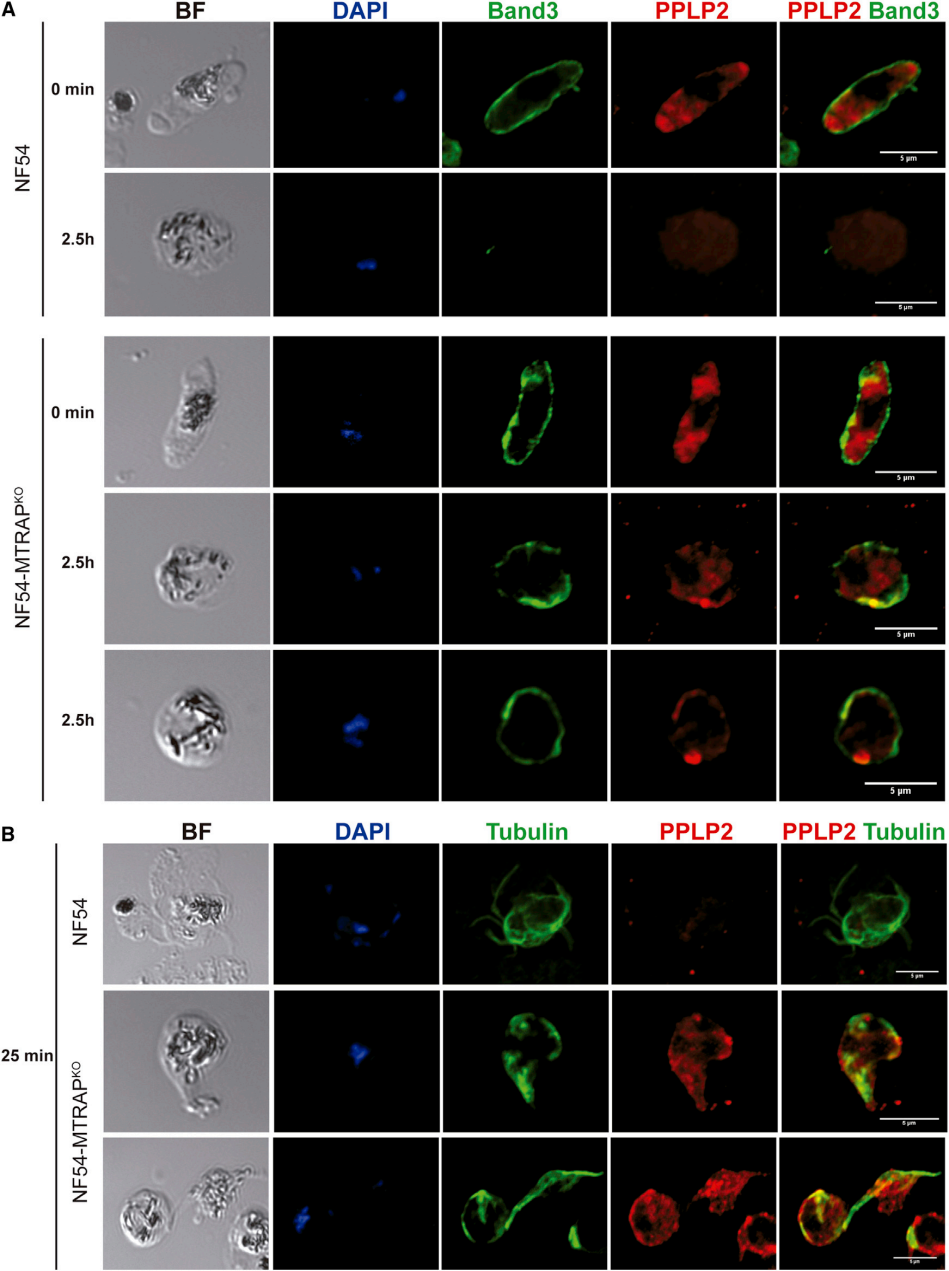
PPLP2 Secretion in *Pf*MTRAP^KO^ Gametocytes (A) Fluorescence microscopy with anti-Band3 (green), anti-PPLP2 (red), and DAPI (blue) in wild-type and MTRAP^KO^ NF54 *P. falciparum* sexual stages matured in vitro nonactivated or 2.5 hr postactivation. BF, brightfield. Scale bar, 5 μm. (B) Fluorescence microscopy with anti-tubulin (green), anti-PPLP2 (red), and DAPI (blue) in wild-type and MTRAP^KO^ NF54 *P. falciparum* sexual stages matured in vitro 25 min postactivation. BF, brightfield. Scale bar, 5 μm.

To follow gamete egress in *Pf*MTRAP^KO^, a knockout was generated in a *P. falciparum* NF54 line, which is more efficient in producing viable gametocytes, using the same plasmids used for generating the knockout in 3D7. *Pf*MTRAP^KO^ had no impact in the time to gametocyte maturation nor in gametocyte yield in cultures. Gametocytes were stained with anti-Band3 for visualization of the EM prior to activation or 2.5 hr after activation. While in wild-type parasites activated gametocytes formed gametes free from a surrounding EM after 2.5 hr, 89.5% of the *Pf*MTRAP^KO^ gametes, relative to the control, were wrapped inside an intact EM (Figure 7A), confirming the *P. berghei* MTRAP^KO^ phenotype.

Last, we tested whether lack of MTRAP might affect secretion of other membrane-lysing effectors upon activation. EM rupture is dependent on the secretion of the *Plasmodium* perforin-like protein 2 (PPLP2), since male and female *P. falciparum* PPLP2^KO^ fail to permeabilize the EM after PVM lysis has occurred (Wirth et al., 2014). In wild-type gametocytes PPLP2 is no longer visible after 2.5 hr activation, likely because it is secreted and lost upon EM lysis. In *Pf*MTRAP^KO^ gametocytes, upon activation PPLP2 staining clearly shifted from a punctate to a periphery-associated pattern, presumably indicating secretion of PPLP2 from internal stores into the PV space but not beyond the unruptured PVM (Figure 7A). This PPLP2 staining pattern was also observed in activated male gametocytes revealed by tubulin staining of formed axonemes. While in exflagellated wild-type male gametocytes PPLP2 had been fully secreted (Figure 7B), activated MTRAP^KO^ male gametocytes formed a thick bundle of flagella, which are motile (Movie S2), surrounded by a periphery-associated PPLP2 staining pattern (Figure 7B). Thus, MTRAP functions critically in the pathway that leads to both male and female gamete egress from the infected erythrocyte.

## Discussion

Proteins of the TRAP family are viewed as bridging extracellular ligands to parasite actin. To date, all members of the family have been shown to play a role during parasite gliding motility and/or host cell invasion (Bargieri et al., 2014). This work shows that at least one member, MTRAP, is involved in another, seemingly unrelated process of rupturing the PVM that surrounds sexual stages inside erythrocytes.

MTRAP was a strong candidate for playing a motor-binding function during merozoite invasion, based on the model that *Plasmodium* merozoite invasion depends on parasite actin (Angrisano et al., 2012, Miller et al., 1979) and the notion that, among parasite surface transmembrane proteins, only TRAP family members were known to bind actin. Our data now indicate that MTRAP acts as a sexual stage protein. Both in *P. berghei* and in *P. falciparum*, immunofluorescence assays using MTRAP antibodies detected the protein in gametocytes. This is in agreement with earlier proteomic studies of *P. falciparum* that identified MTRAP peptides preferentially in gametocyte preparations and clustered MTRAP with a group of proteins predicted to be involved in gametocytogenesis (Silvestrini et al., 2005). Furthermore, *mtrap* gene deletion in *P. berghei* and in *P. falciparum* has no impact on asexual parasite growth in vivo or in vitro, respectively, which fits with the absence of inhibitory effect of MTRAP antibodies (Bartholdson et al., 2012, Uchime et al., 2012) and of recombinant MTRAP (Bartholdson et al., 2012) on *P. falciparum* asexual growth in vitro. Together, these data strongly suggest that MTRAP is not involved in *Plasmodium* merozoite invasion of erythrocytes. The proteins that link the junction to the parasite motor thus remain unknown.

Instead, *mtrap* deletion mutants in both *P. berghei* and *P. falciparum* indicate that male and female mutant gametes fail to egress erythrocytes. Detailed phenotypic characterization of the *P. berghei* mutant indicates that mutant gametes fail to disrupt the PVM, which results in a complete fertilization block in mosquitoes, a phenotype that is partially complemented by an episomally expressed MTRAP. To date, three proteins, which are OB-resident molecules, have been genetically shown to play a role in PVM rupture by activated gametes: PbMDV-1/PEG3 (Ponzi et al., 2009), PbGEST (Talman et al., 2011), and, to a lesser extent, PfDPAP2 (Suárez-Cortés et al., 2016), whereas the partial defect in egress in gametocytes lacking Pfg377 (de Koning-Ward et al., 2008) was no longer observed using a different protocol to measure egress (Suárez-Cortés et al., 2016). None of these proteins, however, displays structural features suggesting a direct role in PVM rupture. Our immunolocalization studies indicate that MTRAP does not convincingly colocalize with any of the OB-resident proteins tested (i.e., Pfg377, MDV-1/PEG3, and DPAP2), suggesting that MTRAP is not stored inside OBs or is in a distinct OB subset. Upon activation, MTRAP then associates with the parasite surface, where it colocalizes with Pfs-230, and remains surface bound after gamete egress.

The finding that MTRAP, a member of the TRAP family of proteins typically associated with parasite gliding motility and host cell invasion, is involved in PVM rupture is unexpected. This, however, does not exclude that MTRAP might still have a function relating to actin/motor based motility. Several lines of evidence are compatible with the involvement of a motor in gamete egress and PVM rupture. It is known that gametocytes possess a trilaminar membrane structure subtended by a layer of structural microtubules that is reminiscent of that of invasive and motile stages (Angrisano et al., 2012, Sinden et al., 1978). It is also known that an F-actin cytoskeleton is concentrated at the ends of the elongating gametocyte, which extends inward along the microtubule cytoskeleton, and is still present in stage V gametocytes (Hliscs et al., 2015). Actin I is the major and ubiquitously expressed actin and is present in both male and female sexual stages, while actin II, a second conventional actin, is specifically expressed by the sexual stages (Deligianni et al., 2011, Rupp et al., 2011, Wesseling et al., 1989). Furthermore, a recent report shows that the subpellicular membrane complex of gametocytes is analogous to the IMC of the parasite motile and invasive stages at the molecular level, since GAP50, GAP45, MTIP, and MyoA are present at the periphery of stages IV and V *P. falciparum* gametocytes (Dearnley et al., 2012). An actomyosin motor role during PVM lysis predicts that cytochalasin D (CytD) and jasplakinolide (Jas), drugs that destabilize actin dynamics, would inhibit egress. In support of this, activated *P. falciparum* gametocytes in the presence of 1 μM of Jas or 10 μM of CytD display an ∼40% and ∼25% inhibition of egress, respectively (data not shown). As such, the notion that actin dynamics are involved in gamete egress certainly warrants further investigation.

Therefore, one possible mechanistic view of the MTRAP-dependent rupture of the gamete-surrounding PVM is that MTRAP links actin in the gamete and ligands on the PVM, which functions as an inverted plasma membrane. In this scenario, actin/motor-mediated displacement/capping of MTRAP along the PPM might lead to the disruption of the PVM. Several lines of indirect evidence fit in well with this hypothesis: (1) surface localization of MTRAP upon gamete activation; (2) a ribbed MTRAP staining pattern seen in mature gametocytes (Figure S6) reminiscent of that seen with GAP50 (Dixon et al., 2012), suggesting that MTRAP might also be, like other members of the TRAP family of proteins, a glideosome-associated protein; (3) patchy distribution of surface-associated MTRAP after activation, suggesting that MTRAP aggregation in the membrane might be part of the activation process; and (4) the recent demonstration, in line with our cytochalasin and jasplakinolide inhibition experiments, that the MTRAP cytoplasmic tail is sufficient to stimulate actin polymerization in vitro (Diaz et al., 2014). Of note, the conformation of recombinant MTRAP (rMTRAP) appears to be a highly extended linear, rod-like protein (2 nm by 33 nm, width by length, respectively) (Uchime et al., 2012), which is in agreement with a putative role of MTRAP in bridging the PPM and PVM. Although Semaphorin-7A was identified as a *P. falciparum* MTRAP binding partner (Bartholdson et al., 2012), it is not involved in MTRAP activity during PVM rupture in *P. berghei*, since WT parasites display normal transmission and thus gamete egress in Semaphorin-7A-deficient mice (Tom Metcalf and Oliver Billker, personal communication). Alternatively, TSRs can also bind heparan sulfates, which might be present at the PVM.

Alternative scenarios are of course possible, although they appear less likely. First, PVM rupture might still depend on MTRAP bridging the PPM to PVM ligands, while MTRAP displacement in the PPM might originate in an actin-independent patching of the protein, as was observed using MTRAP antibodies in samples of 30 s activated *P. falciparum* gametocytes. PVM lysis might also result from some sort of MTRAP-dependent, actin-based gamete motility, which might help disrupt the PVM in the absence of any specific MTRAP-PVM association. Finally, it cannot be excluded that MTRAP might play an indirect role in PVM rupture, such as in signaling or regulation of other effectors. Gametocyte activation is dependent on Ca^2+^ signaling. A Ca^2+^ peak is necessary for male gamete exflagellation, probably through activation of the Calcium-Dependent Protein Kinase 4 (CDPK4), since CDPK4^KO^ parasites fail to exflagellate (Billker et al., 2004). Since MTRAP^KO^ formed beating axonemes, it seems MTRAP is not involved in Ca^2+^ signaling and early steps of gametocyte activation. Another indirect role for MTRAP in PVM lysis might be in regulating the secretions of effectors of membrane rupture. However, our PPLP2 stainings do not favor this view, since PPLP2 is secreted in the MTRAP^KO^. This also suggests that secretion of EM lysis effectors is not dependent on successful PVM rupture.

While the dispensability of MTRAP for asexual growth in the blood excludes MTRAP as a valid target for antimalarial vaccines aimed at preventing merozoite invasion of erythrocytes, MTRAP might still retain potential as a transmission-blocking vaccine. MTRAP function is essential before gamete egress; therefore antibodies will not have access to the target before function. Nonetheless, since MTRAP remains on the surface of egressed gametes, it might serve as a target where bound antibodies might allosterically block gamete function or induce complement-mediated killing. It also remains a possibility that MTRAP might contribute not just to PVM rupture by gametes but also in subsequent steps of zygote formation. Future studies are needed to determine whether specific antibodies to MTRAP can block parasite transmission to mosquitoes.

## Experimental Procedures

### Parasites, Mice, and Mosquitos

*P. berghei* WT ANKA strain and MTRAP^KO^, were maintained in 3-week-old female Wistar rats or 3-week-old female Swiss mice. Mice or rats were infected with *P. berghei* parasites by intraperitoneal or intravenous injections. Parasitemia was followed daily by blood smears or FACS analysis. *Anopheles stephensi* (Sda500 strain) mosquitoes were reared at the Centre for Production and Infection of Anopheles (CEPIA) at the Pasteur Institute. All experiments using rodents were performed in accordance with the guidelines and regulations of the Pasteur Institute and are approved by the Ethical Committee for Animal Experimentation. *P. falciparum* 3D7 and NF54 strains were maintained in RPMI-based media containing O^+^ human erythrocytes at 4% hematocrit and 0.5% AlbuMAX II (Life Technologies) or 10% A^+^ pooled human serum (Interstate Bloodbank), according to established methods (Trager and Jensen, 1976).

### Molecular Cloning and Transfections

To generate the targeting sequence to knockout MTRAP in *P. berghei*, the *mtrap* 5′UTR (553 bp) and 3′UTR (476 bp) were used as homology sequences flanking the hDHFR and mCherry cassettes. The MTRAP complementing plasmid was generated by cloning, in a plasmid bearing the *P. berghei* centromeric sequence CEN-core (Iwanaga et al., 2010), the 5′UTR (1,503 bp), and coding sequence of MTRAP, followed by a heterologous 3′UTR from *trap* (600 bp).

To generate the transfection plasmids for *P. falciparum*, regions of the N-terminal and C-terminal *mtrap* coding sequence, including part of the 5′ and 3′ UTRs, were used as homology regions, which were cloned into the pL6-eGFP CRISPR plasmid on either side of the hDHFR selection cassette (Ghorbal et al., 2014). The guide DNA sequence (GAATGGTCAGAATGTAAAGA) was cloned into the same plasmid using the BtgZI-adaptor site (Ghorbal et al., 2014), resulting in the completed PfMTRAPKO-pL7 plasmid.

*P. berghei* genetic manipulation was performed as described (Lacroix et al., 2011). *P. falciparum* genetic manipulation was performed as described (Fidock and Wellems, 1997).

All primers used for PCR amplification, molecular clonings, and genotyping are described in the Supplemental Information.

### Immunofluorescence Assay

*P. berghei* gametocytes and merozoites were obtained directly from infected mice blood using a Nycodenz gradient (Janse and Waters, 1995). Samples were fixed with 4% paraformaldehyde (PFA) and 0.0075% glutaraldehyde, permeabilized with 0.1% Triton X-100, and blocked with BSA 3% prior to stainings.

*P. falciparum* parasites were obtained from in vitro cultures of the 3D7 or NF54 strains. Synchronous production of gametocytes stages was achieved as described (Fivelman et al., 2007, Lamour et al., 2014). Nonactivated and activated parasites in ookinete medium (RPMI media supplemented with 100 μM xanthurenic acid) were spread on glass slides and fixed with ice-cold methanol.

All antibodies and dilutions used for stainings are described in the the Supplemental Information.

### Electron Microscopy

For analysis of WT and MTRAP^KO^ gametocytes, sexual stages were isolated directly from infected mice blood with at least 0.5% gametocytemia after leucocyte removal (plasmodipur filters, EuroProxima) using a Nycodenz 48% gradient (Janse and Waters, 1995) at 37°C. The cells were with 4% PFA and 1% glutaraldehyde immediately after isolation or after activation in ookinete medium. A detailed description of specimen treatment for EM is provided in the Supplemental Information.

## Author Contributions

Conceptualization, D.Y.B. and R.M.; Methodology, D.Y.B., J.B., G.P., C.L., and R.M.; Validation, T.T. and A.C.; Investigation, D.Y.B., S.T., C.T., A.F.C., A.R., F.H., A.L., U.S., T.T., G.P., and C.L.; Resources, P.S., S.S., T. Tsuboi, C.C., and P.A.; Writing – Original Draft, D.Y.B. and R.M.; Writing – Review and Editing, D.Y.B., P.A., J.B., G.P., C.L., and R.M.; Visualization, D.Y.B.; Project Administration, D.Y.B. and R.M.

## Supplemental Information

Document S1. Supplemental Experimental Procedures and Figures S1–S7

Movie S1. Activated Male *Pb*MTRAPKO Gametocyte Forms Motile Flagella and Remains Trapped inside the Host Cell Related to Figure 3B. Time-lapse light video microscopy of in vitro-activated *P. berghei* MTRAPKO gametocyte. The video plays at 15 frames per second.

Movie S2. Activated Male *Pf*MTRAPKO Gametocyte Forms Motile Flagella and Remains Trapped inside the Host Cell Related to Figure 7. Time-lapse light video microscopy of in vitro-activated NF54 *P. falciparum* MTRAPKO gametocyte. The video plays at 15 frames per second.

Document S2. Article plus Supplemental Information

## References

[bib1] Aikawa M., Miller L.H., Johnson J., Rabbege J. (1978). Erythrocyte entry by malarial parasites. A moving junction between erythrocyte and parasite. J. Cell Biol..

[bib2] Amino R., Thiberge S., Martin B., Celli S., Shorte S., Frischknecht F., Ménard R. (2006). Quantitative imaging of Plasmodium transmission from mosquito to mammal. Nat. Med..

[bib3] Angrisano F., Riglar D.T., Sturm A., Volz J.C., Delves M.J., Zuccala E.S., Turnbull L., Dekiwadia C., Olshina M.A., Marapana D.S. (2012). Spatial localisation of actin filaments across developmental stages of the malaria parasite. PLoS ONE.

[bib4] Bargieri D., Lagal V., Andenmatten N., Tardieux I., Meissner M., Ménard R. (2014). Host cell invasion by apicomplexan parasites: the junction conundrum. PLoS Pathog..

[bib5] Bartholdson S.J., Bustamante L.Y., Crosnier C., Johnson S., Lea S., Rayner J.C., Wright G.J. (2012). Semaphorin-7A is an erythrocyte receptor for P. falciparum merozoite-specific TRAP homolog, MTRAP. PLoS Pathog..

[bib6] Baum J., Richard D., Healer J., Rug M., Krnajski Z., Gilberger T.W., Green J.L., Holder A.A., Cowman A.F. (2006). A conserved molecular motor drives cell invasion and gliding motility across malaria life cycle stages and other apicomplexan parasites. J. Biol. Chem..

[bib7] Billker O., Dechamps S., Tewari R., Wenig G., Franke-Fayard B., Brinkmann V. (2004). Calcium and a calcium-dependent protein kinase regulate gamete formation and mosquito transmission in a malaria parasite. Cell.

[bib8] Boucher L.E., Bosch J. (2015). The apicomplexan glideosome and adhesins - Structures and function. J. Struct. Biol..

[bib9] Combe A., Moreira C., Ackerman S., Thiberge S., Templeton T.J., Ménard R. (2009). TREP, a novel protein necessary for gliding motility of the malaria sporozoite. Int. J. Parasitol..

[bib10] de Koning-Ward T.F., Olivieri A., Bertuccini L., Hood A., Silvestrini F., Charvalias K., Berzosa Díaz P., Camarda G., McElwain T.F., Papenfuss T. (2008). The role of osmiophilic bodies and Pfg377 expression in female gametocyte emergence and mosquito infectivity in the human malaria parasite Plasmodium falciparum. Mol. Microbiol..

[bib11] Dearnley M.K., Yeoman J.A., Hanssen E., Kenny S., Turnbull L., Whitchurch C.B., Tilley L., Dixon M.W. (2012). Origin, composition, organization and function of the inner membrane complex of Plasmodium falciparum gametocytes. J. Cell Sci..

[bib12] Deligianni E., Morgan R.N., Bertuccini L., Kooij T.W., Laforge A., Nahar C., Poulakakis N., Schüler H., Louis C., Matuschewski K., Siden-Kiamos I. (2011). Critical role for a stage-specific actin in male exflagellation of the malaria parasite. Cell. Microbiol..

[bib13] Deligianni E., Morgan R.N., Bertuccini L., Wirth C.C., Silmon de Monerri N.C., Spanos L., Blackman M.J., Louis C., Pradel G., Siden-Kiamos I. (2013). A perforin-like protein mediates disruption of the erythrocyte membrane during egress of Plasmodium berghei male gametocytes. Cell. Microbiol..

[bib14] Dessens J.T., Beetsma A.L., Dimopoulos G., Wengelnik K., Crisanti A., Kafatos F.C., Sinden R.E. (1999). CTRP is essential for mosquito infection by malaria ookinetes. EMBO J..

[bib15] Diaz S.A., Martin S.R., Grainger M., Howell S.A., Green J.L., Holder A.A. (2014). Plasmodium falciparum aldolase and the C-terminal cytoplasmic domain of certain apical organellar proteins promote actin polymerization. Mol. Biochem. Parasitol..

[bib16] Dixon M.W., Dearnley M.K., Hanssen E., Gilberger T., Tilley L. (2012). Shape-shifting gametocytes: how and why does P. falciparum go banana-shaped?. Trends Parasitol..

[bib17] Fidock D.A., Wellems T.E. (1997). Transformation with human dihydrofolate reductase renders malaria parasites insensitive to WR99210 but does not affect the intrinsic activity of proguanil. Proc. Natl. Acad. Sci. USA.

[bib18] Fivelman Q.L., McRobert L., Sharp S., Taylor C.J., Saeed M., Swales C.A., Sutherland C.J., Baker D.A. (2007). Improved synchronous production of Plasmodium falciparum gametocytes in vitro. Mol. Biochem. Parasitol..

[bib19] Frischknecht F., Baldacci P., Martin B., Zimmer C., Thiberge S., Olivo-Marin J.C., Shorte S.L., Ménard R. (2004). Imaging movement of malaria parasites during transmission by Anopheles mosquitoes. Cell. Microbiol..

[bib20] Ghorbal M., Gorman M., Macpherson C.R., Martins R.M., Scherf A., Lopez-Rubio J.J. (2014). Genome editing in the human malaria parasite Plasmodium falciparum using the CRISPR-Cas9 system. Nat. Biotechnol..

[bib21] Gould S.B., Kraft L.G., van Dooren G.G., Goodman C.D., Ford K.L., Cassin A.M., Bacic A., McFadden G.I., Waller R.F. (2011). Ciliate pellicular proteome identifies novel protein families with characteristic repeat motifs that are common to alveolates. Mol. Biol. Evol..

[bib22] Guttery D.S., Roques M., Holder A.A., Tewari R. (2015). Commit and Transmit: Molecular Players in Plasmodium Sexual Development and Zygote Differentiation. Trends Parasitol..

[bib23] Hayton K., Templeton T.J. (2008). Osmiophilic bodies and the odd organelles of alveolates. Mol. Microbiol..

[bib24] Heintzelman M.B. (2015). Gliding motility in apicomplexan parasites. Semin. Cell Dev. Biol..

[bib25] Heiss K., Nie H., Kumar S., Daly T.M., Bergman L.W., Matuschewski K. (2008). Functional characterization of a redundant Plasmodium TRAP family invasin, TRAP-like protein, by aldolase binding and a genetic complementation test. Eukaryot. Cell.

[bib26] Hliscs M., Millet C., Dixon M.W., Siden-Kiamos I., McMillan P., Tilley L. (2015). Organization and function of an actin cytoskeleton in Plasmodium falciparum gametocytes. Cell. Microbiol..

[bib27] Iwanaga S., Khan S.M., Kaneko I., Christodoulou Z., Newbold C., Yuda M., Janse C.J., Waters A.P. (2010). Functional identification of the Plasmodium centromere and generation of a Plasmodium artificial chromosome. Cell Host Microbe.

[bib28] Janse C.J., Waters A.P. (1995). Plasmodium berghei: the application of cultivation and purification techniques to molecular studies of malaria parasites. Parasitol. Today (Regul. Ed.).

[bib29] Jewett T.J., Sibley L.D. (2003). Aldolase forms a bridge between cell surface adhesins and the actin cytoskeleton in apicomplexan parasites. Mol. Cell.

[bib30] Kappe S., Bruderer T., Gantt S., Fujioka H., Nussenzweig V., Ménard R. (1999). Conservation of a gliding motility and cell invasion machinery in Apicomplexan parasites. J. Cell Biol..

[bib31] Kehrer J., Frischknecht F., Mair G.R. (2016). Proteomic analysis of the Plasmodium berghei gametocyte egressome and vesicular bioID of osmiophilic body proteins identifies merozoite TRAP-like Protein (MTRAP) as an essential factor for parasite transmission. Mol. Cell. Proteomics.

[bib32] King C.A. (1988). Cell motility of sporozoan protozoa. Parasitol. Today (Regul. Ed.).

[bib33] Kuehn A., Pradel G. (2010). The coming-out of malaria gametocytes. J. Biomed. Biotechnol..

[bib34] Lacroix C., Giovannini D., Combe A., Bargieri D.Y., Späth S., Panchal D., Tawk L., Thiberge S., Carvalho T.G., Barale J.C. (2011). FLP/FRT-mediated conditional mutagenesis in pre-erythrocytic stages of Plasmodium berghei. Nat. Protoc..

[bib35] Lamour S.D., Straschil U., Saric J., Delves M.J. (2014). Changes in metabolic phenotypes of Plasmodium falciparum in vitro cultures during gametocyte development. Malar. J..

[bib36] Lindner S.E., Miller J.L., Kappe S.H. (2012). Malaria parasite pre-erythrocytic infection: preparation meets opportunity. Cell. Microbiol..

[bib37] Matuschewski K., Nunes A.C., Nussenzweig V., Ménard R. (2002). Plasmodium sporozoite invasion into insect and mammalian cells is directed by the same dual binding system. EMBO J..

[bib38] Miller L.H., Aikawa M., Johnson J.G., Shiroishi T. (1979). Interaction between cytochalasin B-treated malarial parasites and erythrocytes. Attachment and junction formation. J. Exp. Med..

[bib39] Morahan B.J., Wang L., Coppel R.L. (2009). No TRAP, no invasion. Trends Parasitol..

[bib40] Ponzi M., Sidén-Kiamos I., Bertuccini L., Currà C., Kroeze H., Camarda G., Pace T., Franke-Fayard B., Laurentino E.C., Louis C. (2009). Egress of Plasmodium berghei gametes from their host erythrocyte is mediated by the MDV-1/PEG3 protein. Cell. Microbiol..

[bib41] Rangarajan R., Bei A.K., Jethwaney D., Maldonado P., Dorin D., Sultan A.A., Doerig C. (2005). A mitogen-activated protein kinase regulates male gametogenesis and transmission of the malaria parasite Plasmodium berghei. EMBO Rep..

[bib42] Riglar D.T., Whitehead L., Cowman A.F., Rogers K.L., Baum J. (2016). Localisation-based imaging of malarial antigens during erythrocyte entry reaffirms a role for AMA1 but not MTRAP in invasion. J. Cell Sci..

[bib43] Rupp I., Sologub L., Williamson K.C., Scheuermayer M., Reininger L., Doerig C., Eksi S., Kombila D.U., Frank M., Pradel G. (2011). Malaria parasites form filamentous cell-to-cell connections during reproduction in the mosquito midgut. Cell Res..

[bib44] Silvestrini F., Bozdech Z., Lanfrancotti A., Di Giulio E., Bultrini E., Picci L., Derisi J.L., Pizzi E., Alano P. (2005). Genome-wide identification of genes upregulated at the onset of gametocytogenesis in Plasmodium falciparum. Mol. Biochem. Parasitol..

[bib45] Sinden R.E., Canning E.U., Bray R.S., Smalley M.E. (1978). Gametocyte and gamete development in Plasmodium falciparum. Proc. R. Soc. Lond. B Biol. Sci..

[bib46] Sologub L., Kuehn A., Kern S., Przyborski J., Schillig R., Pradel G. (2011). Malaria proteases mediate inside-out egress of gametocytes from red blood cells following parasite transmission to the mosquito. Cell. Microbiol..

[bib47] Steinbuechel M., Matuschewski K. (2009). Role for the Plasmodium sporozoite-specific transmembrane protein S6 in parasite motility and efficient malaria transmission. Cell. Microbiol..

[bib48] Suárez-Cortés P., Sharma V., Bertuccini L., Costa G., Bannerman N.L., Rosa Sannella A., Williamson K., Klemba M., Levashina E.A., Lasonder E., Alano P. (2016). Comparative proteomics and functional analysis reveal a role of P. falciparum osmiophilic bodies in malaria parasite transmission. Mol. Cell. Proteomics.

[bib49] Sultan A.A., Thathy V., Frevert U., Robson K.J., Crisanti A., Nussenzweig V., Nussenzweig R.S., Ménard R. (1997). TRAP is necessary for gliding motility and infectivity of plasmodium sporozoites. Cell.

[bib50] Talman A.M., Lacroix C., Marques S.R., Blagborough A.M., Carzaniga R., Ménard R., Sinden R.E. (2011). PbGEST mediates malaria transmission to both mosquito and vertebrate host. Mol. Microbiol..

[bib51] Tewari R., Dorin D., Moon R., Doerig C., Billker O. (2005). An atypical mitogen-activated protein kinase controls cytokinesis and flagellar motility during male gamete formation in a malaria parasite. Mol. Microbiol..

[bib52] Tibúrcio M., Sauerwein R., Lavazec C., Alano P. (2015). Erythrocyte remodeling by Plasmodium falciparum gametocytes in the human host interplay. Trends Parasitol..

[bib53] Tilley L., Dixon M.W., Kirk K. (2011). The Plasmodium falciparum-infected red blood cell. Int. J. Biochem. Cell Biol..

[bib54] Trager W., Jensen J.B. (1976). Human malaria parasites in continuous culture. Science.

[bib55] Tsuboi T., Takeo S., Iriko H., Jin L., Tsuchimochi M., Matsuda S., Han E.T., Otsuki H., Kaneko O., Sattabongkot J. (2008). Wheat germ cell-free system-based production of malaria proteins for discovery of novel vaccine candidates. Infect. Immun..

[bib56] Uchime O., Herrera R., Reiter K., Kotova S., Shimp R.L., Miura K., Jones D., Lebowitz J., Ambroggio X., Hurt D.E. (2012). Analysis of the conformation and function of the Plasmodium falciparum merozoite proteins MTRAP and PTRAMP. Eukaryot. Cell.

[bib57] Vanderberg J.P., Frevert U. (2004). Intravital microscopy demonstrating antibody-mediated immobilisation of Plasmodium berghei sporozoites injected into skin by mosquitoes. Int. J. Parasitol..

[bib58] Wesseling J.G., Snijders P.J., van Someren P., Jansen J., Smits M.A., Schoenmakers J.G. (1989). Stage-specific expression and genomic organization of the actin genes of the malaria parasite Plasmodium falciparum. Mol. Biochem. Parasitol..

[bib59] Wirth C.C., Pradel G. (2012). Molecular mechanisms of host cell egress by malaria parasites. Int. J. Med. Microbiol..

[bib60] Wirth C.C., Glushakova S., Scheuermayer M., Repnik U., Garg S., Schaack D., Kachman M.M., Weißbach T., Zimmerberg J., Dandekar T. (2014). Perforin-like protein PPLP2 permeabilizes the red blood cell membrane during egress of Plasmodium falciparum gametocytes. Cell. Microbiol..

[bib61] Zieler H., Dvorak J.A. (2000). Invasion in vitro of mosquito midgut cells by the malaria parasite proceeds by a conserved mechanism and results in death of the invaded midgut cells. Proc. Natl. Acad. Sci. USA.

