# Supplementary material for: Plasmodium Merozoite TRAP Family Protein Is Essential for Vacuole Membrane Disruption and Gamete Egress from Erythrocytes
Source: Cell Host Microbe. 2016 Nov 9;20(5):618–30. doi: 10.1016/j.chom.2016.10.015 (PMC5104695; doi:10.1016/j.chom.2016.10.015)
Supplement: Document S1. Supplemental Experimental Procedures and Figures S1–S7 [file mmc1.pdf]

Cell Host & Microbe, Volume *20*

Supplemental Information

Plasmodium Merozoite TRAP Family Protein Is Essential for Vacuole Membrane Disruption and Gamete Egress from Erythrocytes

Daniel Y. Bargieri, Sabine Thiberge, Chwen L. Tay, Alison F. Carey, Alice Rantz, Florian Hischen, Audrey Lorthiois, Ursula Straschil, Pallavi Singh, Shailja Singh, Tony Triglia, Takafumi Tsuboi, Alan Cowman, Chetan Chitnis, Pietro Alano, Jake Baum, Gabriele Pradel, Catherine Lavazec, and Robert Ménard

Material and Methods

Parasites and mosquitos

P. berghei WT ANKA strain, GFP fluorescent (GFP@HSP70) (Ishino et al., 2006), and MTRAP^{KO}, were maintained in 3-week-old female Wistar rats or 3-week-old female Swiss mice. Mice or rats were infected with *P. berghei* parasites by intraperitoneal or intravenous injections. Parasitemia was followed daily by blood smears or FACS analysis. *Anopheles stephensi* (Sda500 strain) mosquitoes were reared at the Centre for Production and Infection of Anopheles (CEPIA) at the Pasteur Institute as described (Thiberge et al., 2007). All experiments using rodents were performed in accordance with the guidelines and regulations of the Pasteur Institute and are approved by the Ethical Committee for Animal Experimentation. *P. falciparum* 3D7 and NF54 strains were maintained in RPMI-based media containing O$^+$ human erythrocytes at 4% haematocrit and 0.5% AlbuMAX II (Life Technologies) or 10% A$^+$ pooled human serum (Interstate Bloodbank), according to established methods (Trager and Jensen, 1976).

Cloning of DNA constructs

To generate the targeting sequence to knockout MTRAP in *P. berghei*, the *mtrap* 5'UTR and 3'UTR were used as homology sequences flanking the hDHFR (de Koning-Ward et al., 2000) and mCherry cassettes. The *mtrap* 5'UTR was amplified from *P. berghei* ANKA genomic DNA with primers forward GGCTGCAGGGGAATTCCGCAAAACTCAAGCATTTTAT (restriction sequences for *Pst*I and *Eco*RI are underlined), and reverse GGGTCGACTTTTCATTTAATTAATCAACACAA (restriction sequence for *Sal*I underlined). The *mtrap* 3'UTR was amplified from *P. berghei* ANKA genomic DNA with primers forward GGGAGCTCGTGTATACAATTTAAGAGAGG (restriction sequence for *Sac*I underlined), and reverse GGGAATTCGTAAACCGTTACCAAAAGTTC (restriction sequence for *Eco*RI underlined). The hDHFR cassette was amplified from a plasmid available in the laboratory with primers forward GGGTCGACTGCAGCCCAGCTTAATTCT (restriction sequence for *Sal*I underlined), and reverse GGCCCGGGAAATTGAAGGAAAAAACATCATT (restriction sequence for *Sma*I underlined). The mCherry cassette (HSP70 promoter) was amplified from plasmid available in the laboratory with primers forward GGCCCGGGGTAATATTTTGTTGGTGAGCT (restriction sequence for *Sma*I underlined) and reverse GGGAGCTCAAAATACCAATAATACCGTTTG (restriction sequence for *Sac*I underlined). The fragments were cloned in the vector pUC18 with the homology regions flanking the hDHFR and mCherry cassettes, generating the plasmid pMTRAP-DCO. The targeting sequence was removed from the vector with *Eco*RI digestion and gel purified for transfection. To construct the MTRAP complementing plasmid, a GFP cassette (HSP70 promoter) was amplified from a plasmid available in the laboratory using primers forward GGCTGCAGGGAAAATATACGTAATATTTTGT (restriction sequence for *Pst*I underlined) and reverse GGGTCGACAAAATACCAATAATACCGTTTG (restriction sequence for *Sal*I underlined),

and cloned in sites *Pst*I and *Sal*I of a pUC18 plasmid bearing the *P. berghei* centromeric sequence CEN-core (Iwanaga et al., 2010) cloned in *Eco*RI, available in the laboratory, resulting in the plasmid pUC-GFP-CC. The 5'UTR and coding sequence of MTRAP were amplified from *P. berghei* ANKA genomic DNA with primers forward GGGTCGACCACATGATATAATAGCTATTC (restriction sequence for *Sal*I underlined) and reverse GGCCCGGGGTATACACTTACTCAGTGCCCC (restriction sequence for *Sma*I underlined), and cloned in sites *Sal*I and *Sma*I of plasmid pUC-GFP-CC, resulting in the plasmid pUC-GFP-MT-CC. The 3'UTR of TRAP was amplified from *P. berghei* ANKA genomic DNA with primers forward GGCCCGGGTTTTAATAAACATATATATCTAGAT (restriction sequence for *Sma*I underlined) and reverse GGGAGCTCTTTTTGTTTCGTAAATTTTAAAATA (restriction sequence for *Sac*I underlined), and cloned in sites *Sma*I and *Sac*I of the plasmid pUC-GFP-MT-CC, resulting in the MTRAP complementing plasmid pUC-GFP-MTRAP-CC.

To generate the transfection plasmids for *P. falciparum*, regions of the N-terminal and C-terminal *mtrap* coding sequence, including part of the 5' and 3' UTRs, were used as homology regions. The N-terminal homology region was amplified from *P. falciparum* 3D7 genomic DNA using the forward primer – CAATGGCCCCTTTCCGCGGTAACATATACATATGAACATGAAC and the reverse primer – TTACAAAATGCTTAAGTCTCATCCTTAATAGAAGAATCAC. The C-terminal homology region was amplified from *P. falciparum* 3D7 genomic DNA using the forward primer – ATTAAATCTAGAATTCCTATGGATTCTATTAAAGAAGAAC and the reverse primer – TTTTACCGTTCCATGGCATTTTTTTCAACATCTCAAGTGG. Restriction sites are shown underlined. The N-terminal and C-terminal homology regions were cloned into the pL6-eGFP CRISPR plasmid on either side of the hDHFR selection cassette (Ghorbal et al., 2014). The pL6-eGFP plasmid was linearised for N-terminal cloning using *Sac*II/*Afl*II restriction sites and for C-terminal cloning using *Eco*RI/*Nco*I restriction sites. The guide DNA sequence (GAATGGTCAGAATGTAAAGA) was cloned into the same plasmid using the BtgZI-adaptor site (Ghorbal et al., 2014), resulting in the completed PfMTRAPKO-pL7 plasmid. All cloning steps were performed using Gibson Assembly (Gibson et al., 2009).

Transfection and selection

P. berghei genetic manipulation was performed as described (Lacroix et al., 2011). *P. berghei* $MTRAP^{KO}$ were generated by double homologous recombination to replace the endogenous *mtrap* coding sequence by a hDHFR cassette and an mCherry fluorescence cassette. The targeting sequence with the two homologous regions flanking the selection cassettes was removed from the plasmid pMTRAP-DCO by *Eco*RI digestion and gel purified using the NucleoSpin Gel and PCR Clean-up kit (Macherey-Nagel) following kit instructions. After transfection of an enriched preparation of *P. berghei* ANKA schizonts and re-injection into mice, mutants were selected with constant treatment with pyrimethamine in drinking water until red fluorescent parasitemia was detected. Drugs were used as described (Janse et al., 2006). Selected red parasites were cloned in mice by limiting dilution and two

clones, B4 and R8, were isolated. Knockout of *mtrap* was confirmed by PCR analysis using primers P1/P2, specific for the WT *mtrap* locus, and P1/P3, specific for integration at the mtrap locus. Primer sequences are P1 - GGTAGATACTGGAAATGATG, P2 – GACAAACGAGTTTATGAGTTT, P3 – GTATACAACTCGCCAAAAAAT. Integration was also confirmed by Southern blotting of total gDNA after digestion with the restriction enzyme *Mfe*I, with a probe hybridizing at the 5'UTR of *mtrap*, amplified with primers forward GATTCAAGTGATGAAAACCCTTTTG and reverse CTCAGTGCCCCAAAATTCA, to recognize the WT and the mutant loci with different sizes. Southern blotting was performed using the DIG easy Hyb kit and DIG wash and block buffer kit from Roche according to manufacturer's protocol.

P. falciparum genetic manipulation was performed as described (Fidock and Wellems, 1997). Briefly, ring stage parasites at 8-10% parasitemia were transfected with 60 µg of the PfMTRAPKO-pL7 plasmid, along with 60 µg of the pUF1-Cas9 plasmid, which expresses the Cas9 endonuclease (Ghorbal et al., 2014). Positive drug selection was performed one day post-transfection using 2.5 nM WR99210 and 1.5 µM DSM1 and maintained until stable parasite growth was achieved. Negative drug selection was then performed using 1 µM 5-fluorocytosine (Sigma Aldrich) in the presence of 2.5 nM WR99210 as previously described (Maier et al., 2006). The transgenic parasites were then cloned by limiting dilution at concentrations of 0.25 parasites/well, 0.5 parasites/well or 1 parasite/well. Three clones were isolated – C3, C8 and C18, and the disruption of *mtrap* was confirmed for each clone by PCR analysis using primers P4/P5, specific for the WT *mtrap* locus, and P4/P6, specific for integration of the targeting sequence at the *mtrap* locus. Primer sequences are P4 – GCGCTCATGTGTATACTTTTATATATG, P5 – CATCATTTTTATCATCATGATCTTC, P6 – GCGACGATGCAGTTTAGCGAACCATGC.

Immunofluorescence assay

For *P. berghei* merozoites, infected mice blood was synchronized in vitro and schizonts were separated by a Nycodenz® 55% gradient as described (Janse and Waters, 1995). *P. berghei* gametocytes were obtained directly from infected mice blood using a Nycodenz® 48 % gradient (Janse and Waters, 1995). Samples were fixed with 4% paraformaldehyde (PFA), 0.0075% glutaraldehyde in PBS for 1 h, permeabilized with 0.1% Triton X-100 in PBS, blocked with BSA 3% in PBS. Stainings were performed with primary rat anti-AMA1 MAb 28G2 (Narum and Thomas, 1994) diluted 1:500, primary rabbit polyclonal antibodies to the *P. berghei* MTRAP peptide YALYNEKNKQSGES (GenScript) diluted 1:500, and primary mouse polyclonal antibodies to the *P. berghei* MDV1-PEG3 protein (Ponzi et al., 2009) followed by secondary Alexa-Fluor-conjugated antibodies (Molecular Probes, 1:500 dilution).

P. falciparum merozoites were obtained from in vitro cultures of the NF54 strain. Parasites were cultivated in vitro under standard conditions using RPMI 1640 medium supplemented with 10% heat-inactivated human serum and human erythrocytes at a 5% haematocrit. Synchronous production of gametocytes stages was achieved as described (Fivelman et al., 2007). Samples were fixed in glass slides with ice-cold methanol pre-activation or 10''and 600''post-activation in ookinete medium and

incubating at 24-26°C (RPMI media supplemented with 100 μM xanthurenic acid), and blocked with BSA 3% in PBS. Stainings were performed with primary rat anti-AMA1 MAb 28G2 (Narum and Thomas, 1994) diluted 1:500, primary rabbit polyclonal antibodies to the *P. falciparum* full-length MTRAP protein produced by wheat germ cell-free *in vitro* translation system (Tsuboi et al., 2008) diluted 1:500, and primary rat anti-Pfg377 (Alano et al., 1995) diluted 1:250 followed by secondary Alexa-Fluor-conjugated antibodies (Molecular Probes, 1:500 dilution). Images were acquired using an Axio Observer Z1 fluorescent microscope (Zeiss).

Alternatively, production of gametocyte stages from wild-type NF54 parasites was achieved as described (Lamour et al., 2014). Stage V gametocytes were purified using Nycoprep 1.077 solution (Axis-Shield) as previously described (Miguel-Blanco et al., 2015). Activation was performed by resuspending purified gametocytes in ookinete medium and incubating at 24-26°C for either 25 minutes or 2.5 hours, as previously described (Delves et al., 2013). Pre-activated and post-activated gametocytes were spread on glass slides and fixed and blocked as described above. Staining was performed with primary rabbit anti-PfMTRAP-TSR (Baum et al., 2006) diluted 1:200, primary rabbit anti-PfMTRAP-tail (Riglar et al., 2016) diluted 1:500, primary mouse anti-GAP45 KN66 (Baum et al., 2006) diluted 1:500, primary mouse anti-Pfs230 diluted 1:500, primary mouse anti-Tubulin diluted 1:500, primary rabbit anti-PPLP2 diluted 1:100, primary mouse anti-DPAP2 diluted 1:50, primary rat anti-Band3 (BRAC66) diluted 1:250, followed by secondary Alexa-Fluor-conjugated antibodies (Life Technologies, 1:500 dilution). Confocal images were acquired using a Zeiss LSM 510 Laser scanning confocal microscope.

Western blot analysis

For Western blot analysis, *P. berghei* total blood stages or isolated gametocytes, or transgenic schizont-stage *P. falciparum* parasites were extracted from *in vitro* blood-stage cultures by saponin lysis. Saponin pellets were lysed in 4%SDS/50 mM Tris-Cl pH 7.4/150 mM NaCl/5 mM EDTA and boiled at 95°C for 5 minutes. For NF54 pre- and post-activated gametocytes, the Nycoprep-purified gametocytes were directly lysed and boiled as described above. Lysates were resuspended in reducing 2X Laemmli sample buffer and resolved by SDS-PAGE. Proteins of interest were detected using the following antibodies diluted in 1% skim milk/0.1% Tween-20/PBS: primary rabbit antiPbMTRAP diluted 1:1000, anti-*Plasmodium* aldolase-HRP (Abcam ab38905) diluted 1:2000, primary rabbit anti-PfMTRAP-tail (Riglar et al., 2016) diluted 1:500, primary mouse PfActin 5H3 diluted 1:500, followed by secondary HRP-conjugated antibodies (Sigma-Aldrich).

P. falciparum growth assay

P. falciparum blood-stage culture was diluted to produce a suspension at 2% haematocrit and 2% parasitemia. A 10 μL aliquot of the parasite suspension was fixed in 2% paraformaldehyde/0.2% gluteraldehyde/PBS for 45 minutes at 4°C. The fixed cells ('Day 0') were kept in 50 μL PBS until further

use. The remaining parasite suspension was added into a 96-well round-bottomed plate at 100 µL volume per well. After 48 hours incubation under standard culture conditions, a 10 µL aliquot was again removed from the plate and fixed as above ('Day 2'). The cells in the plate were then diluted 1:10 or 1:20 with fresh red blood cells (2% haematocrit). This was repeated every 48 hours until 'Day 10' cells were collected. Fixed cells were permeabilised in 0.3% Triton X-100/PBS for 10 minutes at room temperature (RT) and stained with SYBR Green I (Life Technologies)/PBS, at a concentration of 1:5000, for 45 minutes at RT. This was followed by acquisition on a flow cytometer and parasitemia was determined by SYBR Green I fluorescence as measured by the flow cytometer. All experiments were carried out in triplicate and the data is presented as mean ± standard deviation.

Mosquito infection

An. stephensi mosquitos were kept and infected as previously described (Sinden et al., 2002; Thiberge et al., 2007). Oocyst infection in mosquito midgut was verified 7 days after mosquito infection by removal of the midgut with the aid of a syringe needle and a forceps, staining of the midgut with 1% solution of mercurochrome in PBS (Sinden et al., 2002), and observation under light microscope. In some cases the midguts were directly observed under a fluorescence microscope (Axio Observer Z1 fluorescent microscope, Zeiss).

Gametocyte in vitro activation assays and ookinete culture

P. berghei gametocyte activation in vitro was achieved by collecting 5 µl of infected mice blood with at least 0.5 % gametocytemia and immediately mixing with 10 µl of PBS at RT. One third of the mixture was put in a glass slide, covered with a coverslip, and activation was observed under light microscopy for 20 minutes for videomicroscopy (Axio Observer Z1 fluorescent microscope, Zeiss) or counting of exflagellation centres. *P. berghei* ookinete cultures were performed as described (Ramakrishnan et al., 2013).

Electron microscopy

For analysis of WT and MTRAP^{KO} gametocytes, sexual stages were isolated directly from infected mice blood with at least 0.5 % gametocytemia after leucocyte removal (plasmodipur filters, EuroProxima) using a Nycodenz® 48% gradient (Janse and Waters, 1995) at 37 ºC to avoid gametocyte activation. The cells were washed in RPMI at 37 ºC and separated in two tubes pre heated at 37 ºC. One tube was immediately fixed with 4 % PFA and 1 % glutaraldehyde. The second tube was activated with ookinete medium at RT (Ramakrishnan et al., 2013) for 15 minutes prior to fixation. Specimens were post-fixed in 1% osmium tetroxide and 1.5% K3Fe(CN)6 in PBS for 2 h at RT, followed by incubation in 0.5% uranyl acetate for 1 h. The fixed cultures were dehydrated in increasing concentrations of ethanol and then incubated for 1 h in propylene oxide, followed by another incubation step for 1 h in a 1:1 mixture of propylene oxide and Epon (Electron Microscopy Sciences). Specimens were subsequently embedded

in Epon at 60°C for 2 d. Post-contrasting of ultrasections included sequential incubation steps with 2% uranyl acetate for 20 min and 0.2% lead citrate for 7 min and rinsing of the ultrasections in A. bidest for 5x 30 s. Photographs were taken with a Zeiss EM10 transmission electron microscope and scanned images were processed using Adobe Photoshop CS software.

Supplemental References

Alano, P., Read, D., Bruce, M., Aikawa, M., Kaido, T., Tegoshi, T., Bhatti, S., Smith, D.K., Luo, C., Hansra, S., *et al.* (1995). COS cell expression cloning of Pfg377, a Plasmodium falciparum gametocyte antigen associated with osmiophilic bodies. Mol Biochem Parasitol *74*, 143-156.

Baum, J., Richard, D., Healer, J., Rug, M., Krnajski, Z., Gilberger, T.W., Green, J.L., Holder, A.A., and Cowman, A.F. (2006). A conserved molecular motor drives cell invasion and gliding motility across malaria life cycle stages and other apicomplexan parasites. J Biol Chem *281*, 5197-5208.

de Koning-Ward, T.F., Fidock, D.A., Thathy, V., Menard, R., van Spaendonk, R.M., Waters, A.P., and Janse, C.J. (2000). The selectable marker human dihydrofolate reductase enables sequential genetic manipulation of the Plasmodium berghei genome. Mol Biochem Parasitol *106*, 199-212.

Delves, M.J., Ruecker, A., Straschil, U., Lelievre, J., Marques, S., Lopez-Barragan, M.J., Herreros, E., and Sinden, R.E. (2013). Male and female Plasmodium falciparum mature gametocytes show different responses to antimalarial drugs. Antimicrob Agents Chemother *57*, 3268-3274.

Fidock, D.A., and Wellems, T.E. (1997). Transformation with human dihydrofolate reductase renders malaria parasites insensitive to WR99210 but does not affect the intrinsic activity of proguanil. Proc Natl Acad Sci U S A *94*, 10931-10936.

Fivelman, Q.L., McRobert, L., Sharp, S., Taylor, C.J., Saeed, M., Swales, C.A., Sutherland, C.J., and Baker, D.A. (2007). Improved synchronous production of Plasmodium falciparum gametocytes in vitro. Mol Biochem Parasitol *154*, 119-123.

Ghorbal, M., Gorman, M., Macpherson, C.R., Martins, R.M., Scherf, A., and Lopez-Rubio, J.J. (2014). Genome editing in the human malaria parasite Plasmodium falciparum using the CRISPR-Cas9 system. Nat Biotechnol *32*, 819-821.

Gibson, D.G., Young, L., Chuang, R.Y., Venter, J.C., Hutchison, C.A., 3rd, and Smith, H.O. (2009). Enzymatic assembly of DNA molecules up to several hundred kilobases. Nat Methods *6*, 343-345.

Ishino, T., Orito, Y., Chinzei, Y., and Yuda, M. (2006). A calcium-dependent protein kinase regulates Plasmodium ookinete access to the midgut epithelial cell. Mol Microbiol *59*, 1175-1184.

Iwanaga, S., Khan, S.M., Kaneko, I., Christodoulou, Z., Newbold, C., Yuda, M., Janse, C.J., and Waters, A.P. (2010). Functional identification of the Plasmodium centromere and generation of a Plasmodium artificial chromosome. Cell host & microbe *7*, 245-255.

Janse, C.J., Ramesar, J., and Waters, A.P. (2006). High-efficiency transfection and drug selection of genetically transformed blood stages of the rodent malaria parasite Plasmodium berghei. Nat Protoc *1*, 346-356.

Janse, C.J., and Waters, A.P. (1995). Plasmodium berghei: the application of cultivation and purification techniques to molecular studies of malaria parasites. Parasitology today *11*, 138-143.

Lacroix, C., Giovannini, D., Combe, A., Bargieri, D.Y., Spath, S., Panchal, D., Tawk, L., Thiberge, S., Carvalho, T.G., Barale, J.C., *et al.* (2011). FLP/FRT-mediated conditional mutagenesis in pre-erythrocytic stages of Plasmodium berghei. Nat Protoc *6*, 1412-1428.

Lamour, S.D., Straschil, U., Saric, J., and Delves, M.J. (2014). Changes in metabolic phenotypes of Plasmodium falciparum in vitro cultures during gametocyte development. Malar J *13*, 468.

Maier, A.G., Braks, J.A., Waters, A.P., and Cowman, A.F. (2006). Negative selection using yeast cytosine deaminase/uracil phosphoribosyl transferase in Plasmodium falciparum for targeted gene deletion by double crossover recombination. Mol Biochem Parasitol *150*, 118-121.

Miguel-Blanco, C., Lelievre, J., Delves, M.J., Bardera, A.I., Presa, J.L., Lopez-Barragan, M.J., Ruecker, A., Marques, S., Sinden, R.E., and Herreros, E. (2015). Imaging-based high-throughput screening assay to identify new molecules with transmission-blocking potential against Plasmodium falciparum female gamete formation. Antimicrob Agents Chemother *59*, 3298-3305.

Narum, D.L., and Thomas, A.W. (1994). Differential localization of full-length and processed forms of PF83/AMA-1 an apical membrane antigen of Plasmodium falciparum merozoites. Mol Biochem Parasitol *67*, 59-68.

Ponzi, M., Siden-Kiamos, I., Bertuccini, L., Curra, C., Kroeze, H., Camarda, G., Pace, T., Franke-Fayard, B., Laurentino, E.C., Louis, C., *et al.* (2009). Egress of Plasmodium berghei gametes from their host erythrocyte is mediated by the MDV-1/PEG3 protein. Cell Microbiol *11*, 1272-1288.

Ramakrishnan, C., Delves, M.J., Lal, K., Blagborough, A.M., Butcher, G., Baker, K.W., and Sinden, R.E. (2013). Laboratory maintenance of rodent malaria parasites. Methods Mol Biol *923*, 51-72.

Riglar, D.T., Whitehead, L., Cowman, A.F., Rogers, K.L., and Baum, J. (2016). Localisation-based imaging of malarial antigens during erythrocyte entry reaffirms a role for AMA1 but not MTRAP in invasion. J Cell Sci *129*, 228-242.

Sinden, R.E., Butcher, G.A., and Beetsma, A.L. (2002). Maintenance of the Plasmodium berghei life cycle. Methods Mol Med *72*, 25-40.

Thiberge, S., Blazquez, S., Baldacci, P., Renaud, O., Shorte, S., Menard, R., and Amino, R. (2007). In vivo imaging of malaria parasites in the murine liver. Nat Protoc *2*, 1811-1818.

Trager, W., and Jensen, J.B. (1976). Human malaria parasites in continuous culture. Science *193*, 673-675.

Tsuboi, T., Takeo, S., Iriko, H., Jin, L., Tsuchimochi, M., Matsuda, S., Han, E.T., Otsuki, H., Kaneko, O., Sattabongkot, J., *et al.* (2008). Wheat germ cell-free system-based production of malaria proteins for discovery of novel vaccine candidates. Infect Immun *76*, 1702-1708.

Supplementary Figure 1

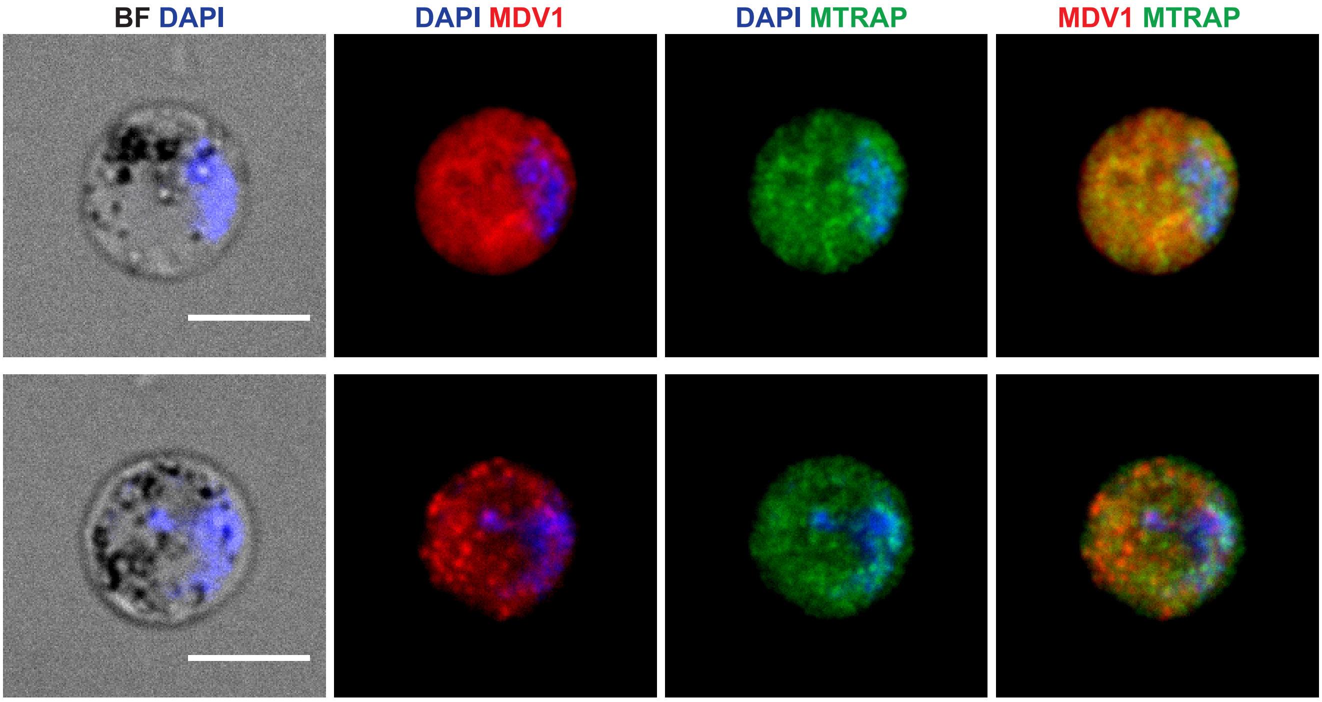

MTRAP staining in *P. berghei* gametocytes - related to Figure 1f
Fluorescence microscopy with anti-PbMTRAP (green), anti-MDV-1/PEG3 (red) and DAPI (blue) in wild type non-activated *P. berghei* sexual stages isolated from infected mouse blood. BF: bright field. Scale bar: 5 μm.

Supplementary Figure 2

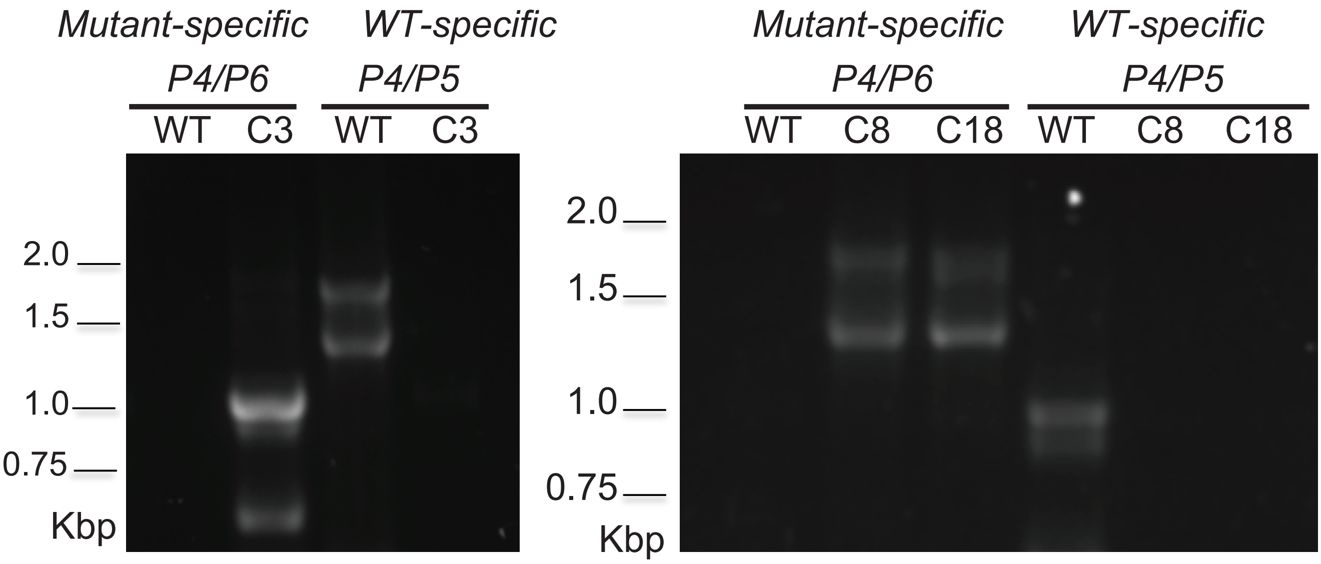

PCR genotype of *Pf*MTRAP^{KO} - related to Figure 5

PCR analysis of the mtrap loci in wild type (WT) or mutant (C3, C8 and C18) *P. falciparum* parasites. P4/P5 pair of primers is specific to the wild type locus, and P4/P6 pair is specific to integration of the targeting sequence.

Supplementary Figure 3

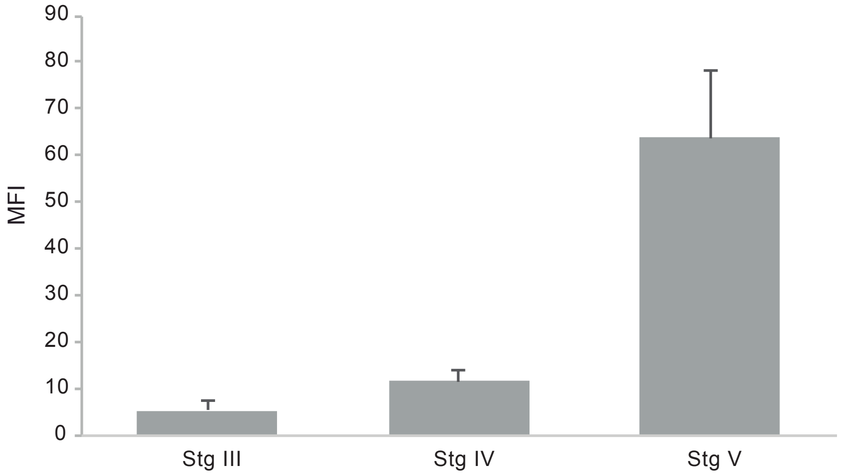

MFI of MTRAP staining in *P. falciparum* gametocytes - related to Figure 6a

Mean fluorescent intensity (MFI) in arbitrary units of MTRAP staining of 3D7 *P. falciparum* gametocytes in stages III, IV and V of maturation. The results are shown as mean +/- SD and are representative of two independent experiments.

Supplementary Figure 4

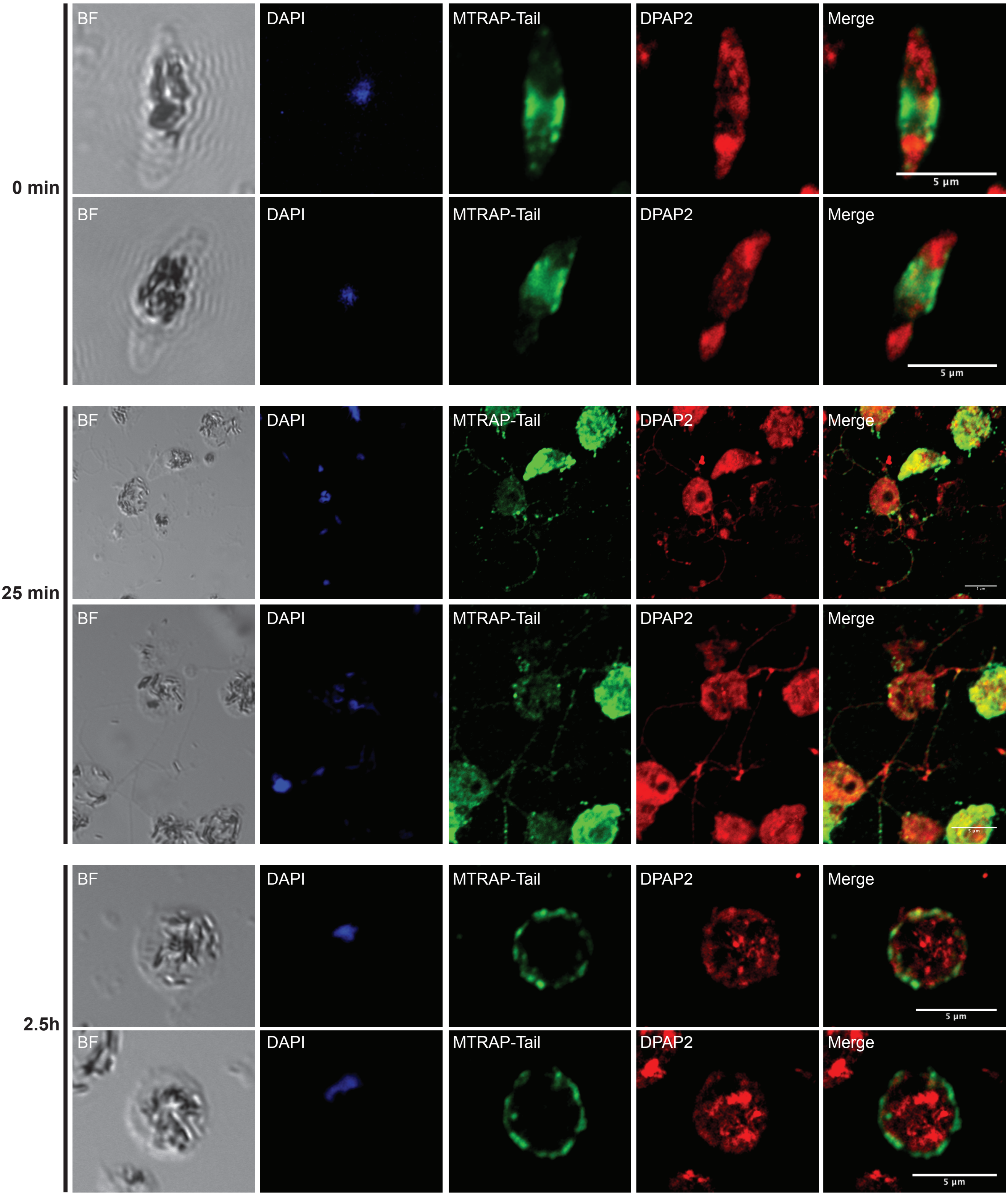

DPAP2 and MTRAP staining in *P. falciparum* gametocytes - related to figure 6
Fluorescence microscopy of non-activated, activated for 25 min or for 2.5 hours wild type 3D7 *P. falciparum* gametocytes with anti-PfMTRAP-Tail (green), anti-DPAP2 (red) and DAPI (blue). BF: bright field. Scale bar: 5 µm.

Supplementary Figure 5

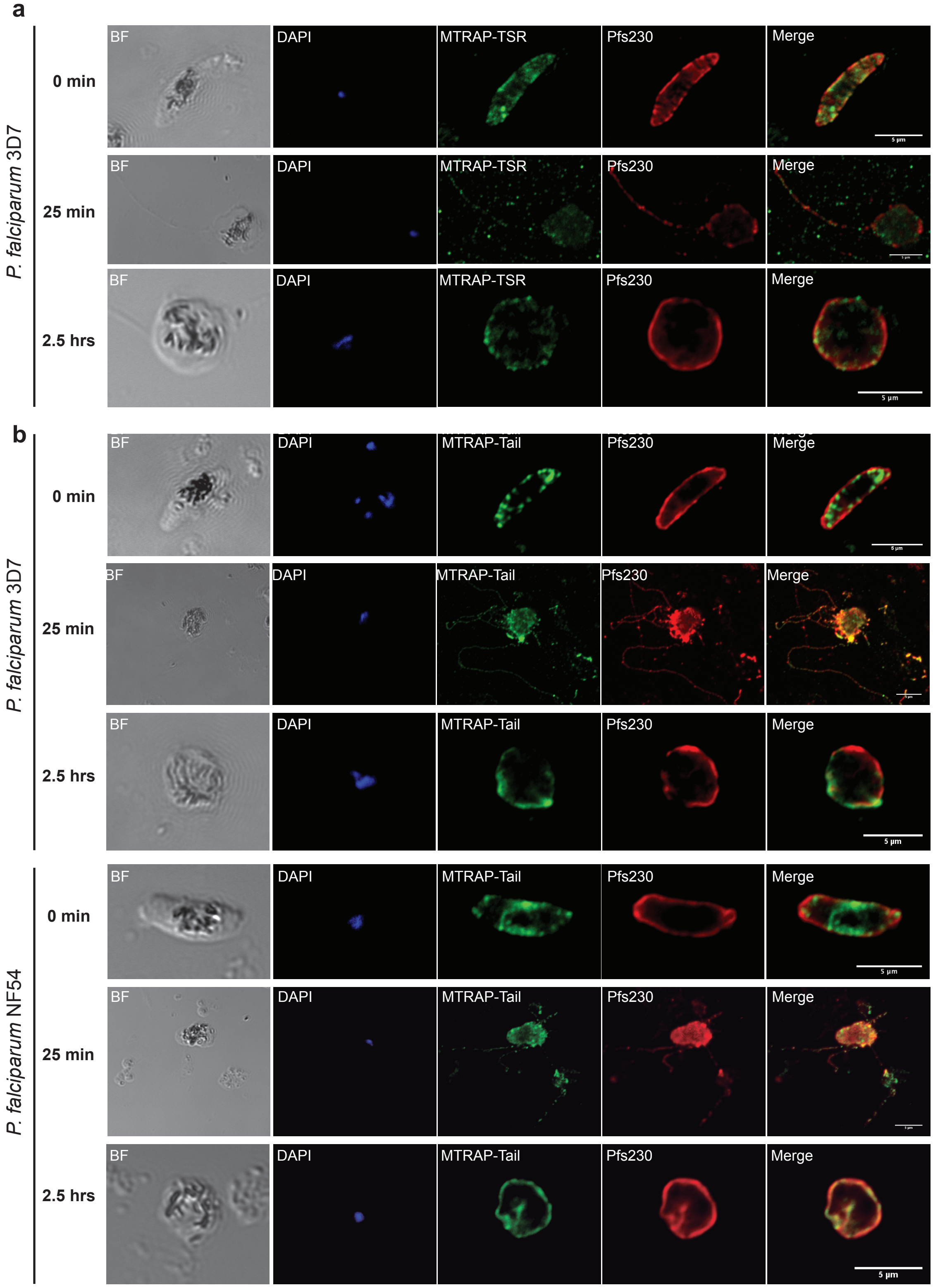

Pfs230 and MTRAP staining in *P. falciparum* gametocytes - related to figure 6

(A) Fluorescence microscopy of non-activated, activated for 25 min or for 2.5 hours wild type 3D7 *P. falciparum* 3D7 gametocytes with anti-PfMTRAP-TSR (green), anti--Pfs230 (red) and DAPI (blue). BF: bright field. Scale bar: 5 µm.

(B) Fluorescence microscopy of non-activated, activated for 25 min or for 2.5 hours wild type 3D7 *P. falciparum* 3D7 or NF54 gametocytes with anti-PfMTRAP-Tail (green), anti-Pfs230 (red) and DAPI (blue). BF: bright field. Scale bar: 5 µm.

Supplementary Figure 6

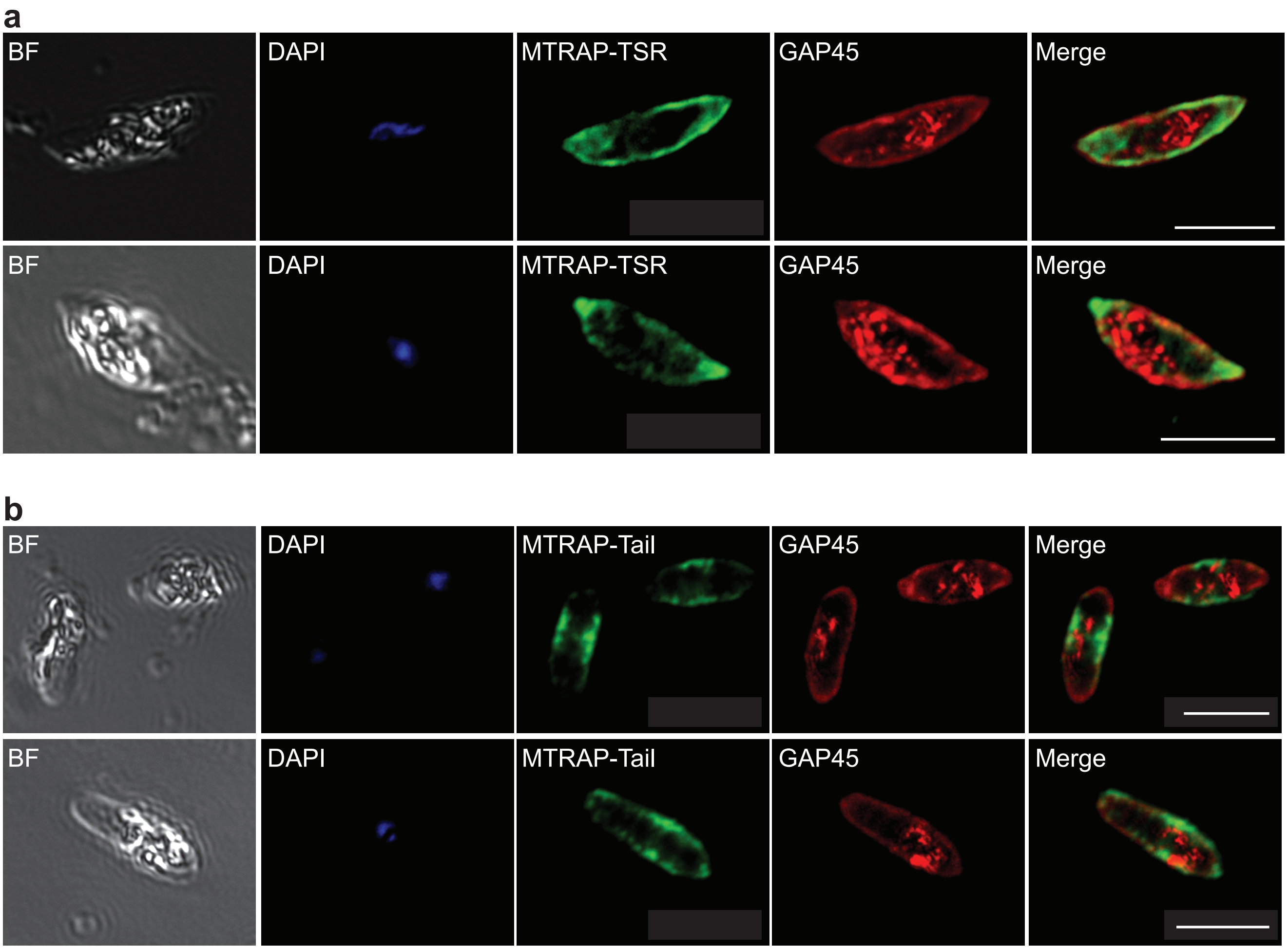

GAP45 and MTRAP staining in *P. falciparum* gametocytes - related to figure 6

(A) Fluorescence microscopy of non-activated wild type *P. falciparum* 3D7 gametocytes with anti-PfMTRAP-TSR (green), anti-GAP45 (red) and DAPI (blue). BF: bright field. Scale bar: 5 µm.

(B) Fluorescence microscopy of non-activated wild type *P. falciparum* 3D7 gametocytes with anti-PfMTRAP-Tail (green), anti-GAP45 (red) and DAPI (blue). BF: bright field. Scale bar: 5 µm.

Supplementary Figure 7

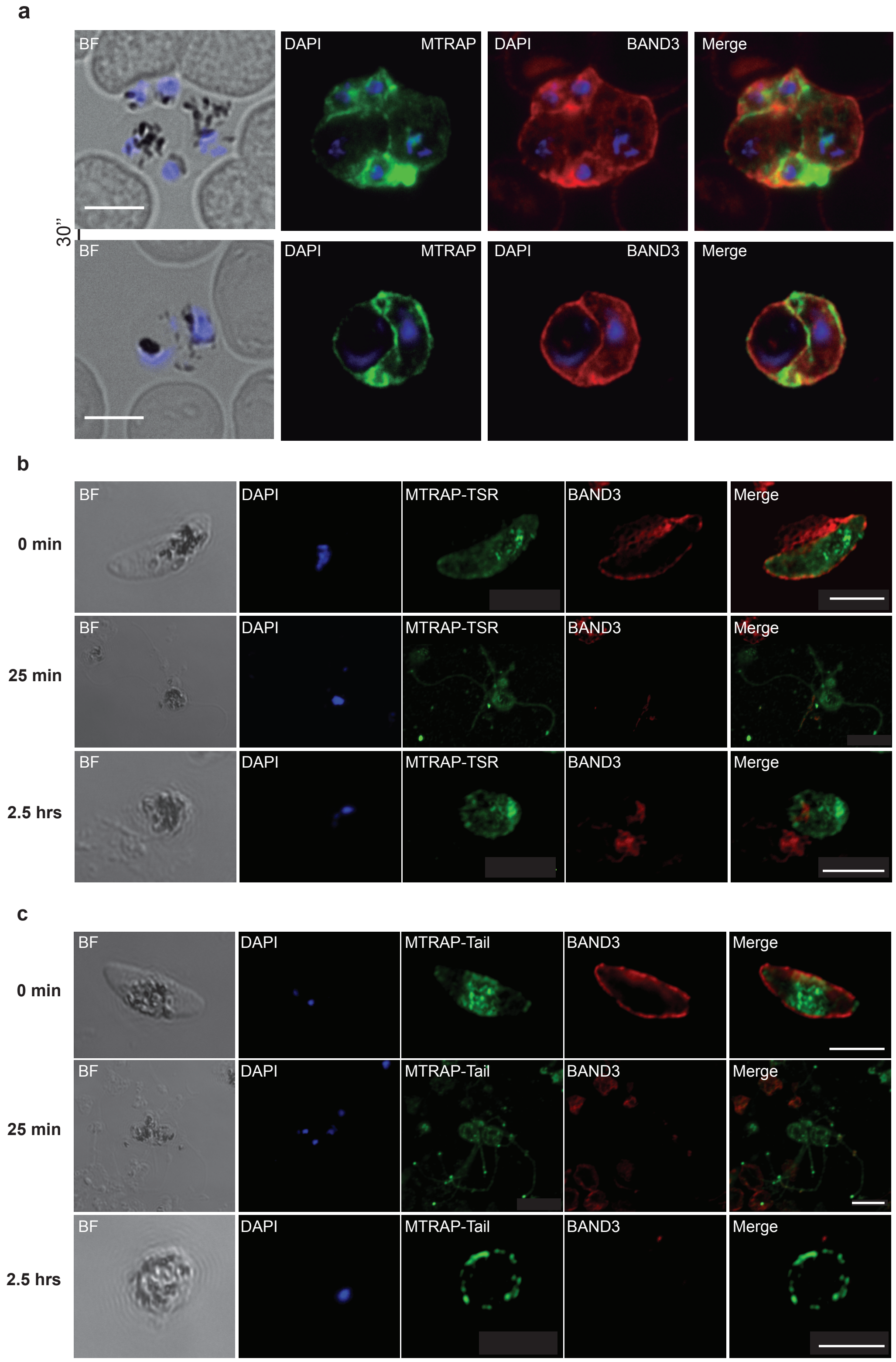

MTRAP staining in *P. falciparum* gametocytes through activation - related to Figure 6

(A) Fluorescence microscopy with anti-MTRAP (green), anti-Band3 (red) and DAPI (blue) in wild type stage V 3D7 *P. falciparum* sexual stages matured in vitro and activated for 30 seconds in ookinete medium. BF: bright field. Scale bar: 5 µm.

(B) Fluorescence microscopy of non-activated, activated for 25 min or for 2.5 hours wild type 3D7 *P. falciparum* gametocytes with anti-PfMTRAP-TSR (green), anti-Band3 (red) and DAPI (blue). BF: bright field. Scale bar: 5 µm.

(C) Fluorescence microscopy of non-activated, activated for 25 min or for 2.5 hours wild type 3D7 *P. falciparum* gametocytes with anti-PfMTRAP-Tail (green), anti-Band3 (red) and DAPI (blue). BF: bright field. Scale bar: 5 µm.

Supplementary movie legends

Supplementary movie 1. Activated male *Pb*MTRAP^{KO} gametocyte forms motile flagella and remains trapped inside the host cell – related to Figure 3b

Time-lapse light video microscopy of *in vitro* activated *P. berghei* MTRAP^{KO} gametocyte. The video plays at 15 frames per second.

Supplementary movie 2. Activated male *Pf*MTRAP^{KO} gametocyte forms motile flagella and remains trapped inside the host cell – related to Figure 7

Time-lapse light video microscopy of *in vitro* activated NF54 *P. falciparum* MTRAP^{KO} gametocyte. The video plays at 15 frames per second.
