# Supplementary figures and images for: Plasmodium Merozoite TRAP Family Protein Is Essential for Vacuole Membrane Disruption and Gamete Egress from Erythrocytes
Source: Cell Host Microbe. 2016 Nov 9;20(5):618–30. doi: 10.1016/j.chom.2016.10.015 (PMC5104695; doi:10.1016/j.chom.2016.10.015)

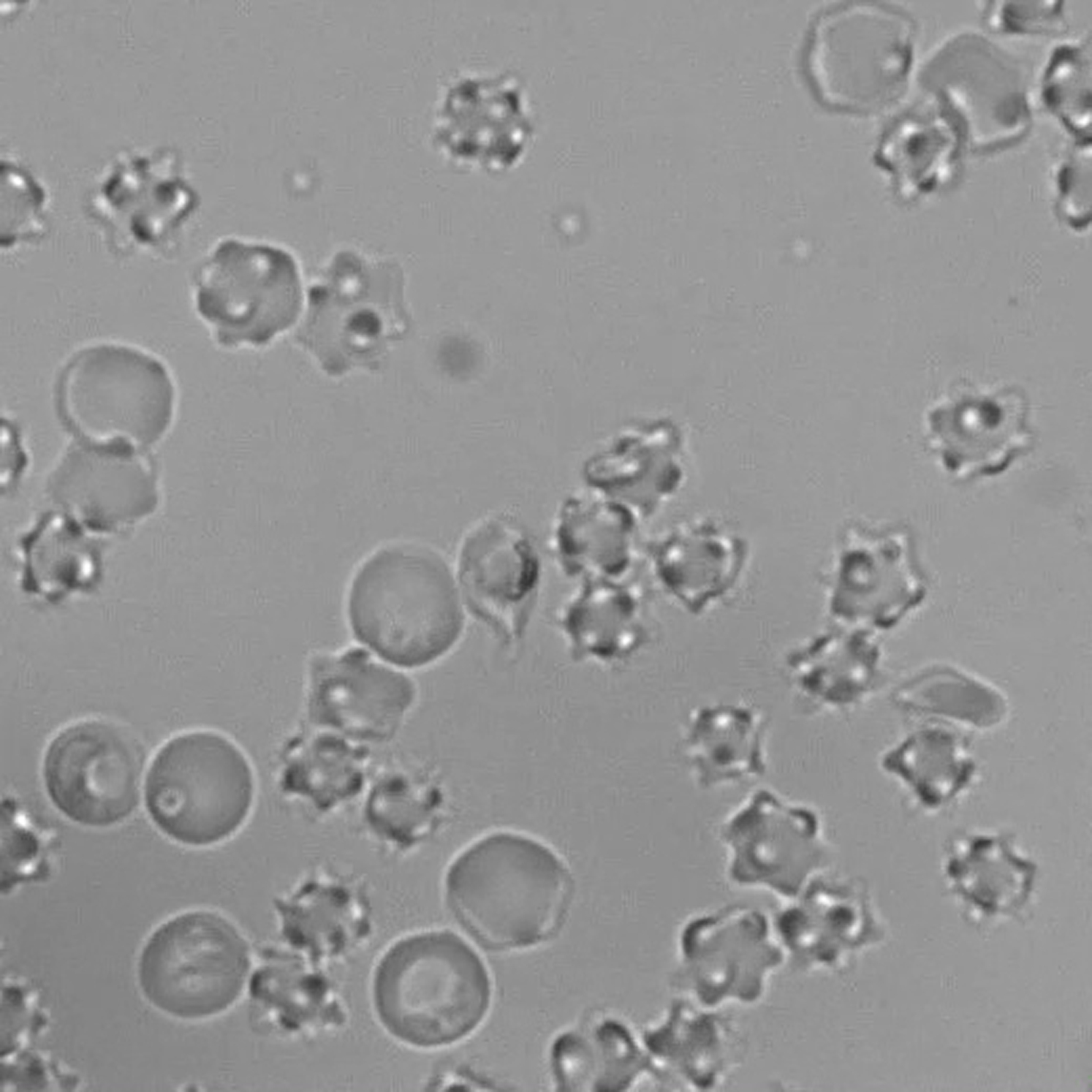

Supplement: Movie S1. Activated Male PbMTRAPKO Gametocyte Forms Motile Flagella and Remains Trapped inside the Host Cell — Related to Figure 3B. Time-lapse light video microscopy of in vitro-activated P. berghei MTRAPKO gametocyte. The video plays at 15 frames per second. [file mmc2.jpg]

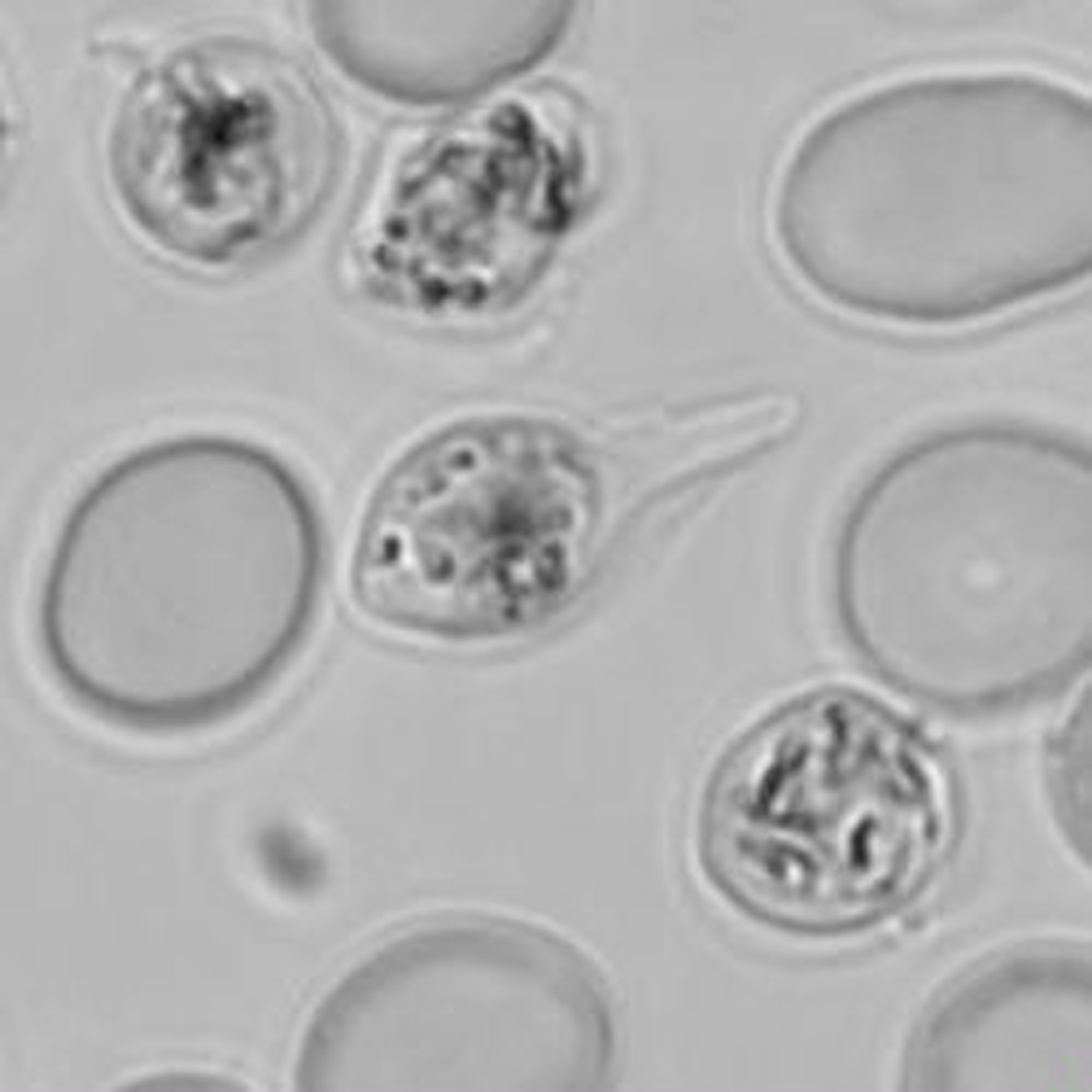

Supplement: Movie S2. Activated Male PfMTRAPKO Gametocyte Forms Motile Flagella and Remains Trapped inside the Host Cell — Related to Figure 7. Time-lapse light video microscopy of in vitro-activated NF54 P. falciparum MTRAPKO gametocyte. The video plays at 15 frames per second. [file mmc3.jpg]
